# The Influence of Glacial Cover on Riverine Silicon and Iron Exports in Chilean Patagonia

**DOI:** 10.1029/2020GB006611

**Published:** 2020-12-17

**Authors:** Helena V. Pryer, Jon R. Hawkings, Jemma L. Wadham, Laura F. Robinson, Katharine R. Hendry, Jade E. Hatton, Anne M. Kellerman, Sebastien Bertrand, Beatriz Gill‐Olivas, Matthew G. Marshall, Richard A. Brooker, Giovanni Daneri, Vreni Häussermann

**Affiliations:** ^1^ Bristol Glaciology Centre, Department of Geographical Sciences University of Bristol Bristol UK; ^2^ School of Earth Sciences University of Bristol Bristol UK; ^3^ Department of Earth, Ocean and Atmospheric Sciences Florida State University Tallahassee FL USA; ^4^ German Research Centre for Geosciences GFZ Potsdam Germany; ^5^ Renard Centre of Marine Geology Ghent University Ghent Belgium; ^6^ Centro de Investigación en Ecosistemas de la Patagonia Coyhaique Chile; ^7^ COPAS Sur‐Austral Universidad de Concepción Concepción Chile; ^8^ Huinay Scientific Field Station Pontificia Universidad Católica de Valparaíso Valparaíso Chile

**Keywords:** Patagonia, silicon, iron, rivers, nutrients, glaciers

## Abstract

Glaciated environments have been highlighted as important sources of bioavailable nutrients, with inputs of glacial meltwater potentially influencing productivity in downstream ecosystems. However, it is currently unclear how riverine nutrient concentrations vary across a spectrum of glacial cover, making it challenging to accurately predict how terrestrial fluxes will change with continued glacial retreat. Using 40 rivers in Chilean Patagonia as a unique natural laboratory, we investigate how glacial cover affects riverine Si and Fe concentrations, and infer how exports of these bioessential nutrients may change in the future. Dissolved Si (as silicic acid) and soluble Fe (<0.02 μm) concentrations were relatively low in glacier‐fed rivers, whereas concentrations of colloidal‐nanoparticulate (0.02–0.45 μm) Si and Fe increased significantly as a function of glacial cover. These colloidal‐nanoparticulate phases were predominately composed of aluminosilicates and Fe‐oxyhydroxides, highlighting the need for size‐fractionated analyses and further research to quantify the lability of colloidal‐nanoparticulate species. We also demonstrate the importance of reactive particulate (>0.45 μm) phases of both Si and Fe, which are not typically accounted for in terrestrial nutrient budgets but can dominate riverine exports. Dissolved Si and soluble Fe yield estimates showed no trend with glacial cover, suggesting no significant change in total exports with continued glacial retreat. However, yields of colloidal‐nanoparticulate and reactive sediment‐bound Si and Fe were an order of magnitude greater in highly glaciated catchments and showed significant positive correlations with glacial cover. As such, regional‐scale exports of these phases are likely to decrease as glacial cover disappears across Chilean Patagonia, with potential implications for downstream ecosystems.

## Key Points

- Si and Fe concentrations from 40 rivers in Chilean Patagonia reveal the impact of glacial cover on terrestrial nutrient cycling
- Colloidal and reactive particulate phases of Si and Fe are elevated in glacier‐fed rivers and dominate export budgets
- Riverine exports of Si and Fe are likely to significantly change with continued glacial retreat, which may impact productivity in downstream ecosystems

## 1 Introduction

One of the most visible effects of climate change is melting of the Earth's cryosphere, with the majority of glaciers currently losing mass at increasingly rapid rates (Zemp et al., 2019). The physical impacts of deglaciation, such as reduced global albedo (Budyko, 1969; Pistone et al., 2014) and sea‐level rise (Gardner et al., 2013; Zemp et al., 2019), are relatively well documented. However, the implications of enhanced glacial retreat on biogeochemical cycles are still not well understood, despite potentially critical impacts for coastal ecosystems, the carbon cycle, and global climate (Wadham et al., 2019). Until two decades ago, ice sheets and glaciers were thought to be inert, passive components of biogeochemical cycles. However, glacial environments are now recognized as active biomes (Anesio & Laybourn‐Parry, 2012; Anesio et al., 2017; Hodson et al., 2008) and hot spots for biogeochemical weathering (Wadham et al., 2010), with reactions between glacial meltwaters and finely ground rock flour liberating bioavailable nutrient phases from the bedrock (Wadham et al., 2019). As such, glacial systems have been highlighted as natural factories for lithogenic nutrients, with meltwaters potentially sustaining productivity in downstream ecosystems (Hawkings et al., 2014, 2015; Wadham et al., 2019).

Glacial weathering processes have been identified to be particularly important in the generation of potentially bioavailable iron (Fe) (Bhatia et al., 2013; Hawkings et al., 2014; Li et al., 2019; Raiswell et al., 2018; Schroth et al., 2011). Fe is released from the bedrock by a series of microbially mediated weathering reactions, including silicate dissolution, sulfide oxidation, and iron reduction, all of which occur in the subglacial environment (Mikucki et al., 2009; Nixon et al., 2017; Tranter et al., 2002). Since Fe is the main limiting nutrient in 30–50% of the ocean (Boyd et al., 2007; Martin et al., 1990; Moore et al., 2013), changes in terrestrial Fe fluxes have the potential to affect primary productivity, with implications for oceanic food chains and carbon sequestration (Jickells et al., 2005; Tagliabue et al., 2017). In the Southern Ocean, the delivery of ice‐rafted Fe‐rich terrigenous material has been identified as a key control on primary productivity, with phytoplankton blooms found in the wake of icebergs (Duprat et al., 2016; Schwarz & Schodlok, 2009; Smith et al., 2007). In addition, glacial meltwater from the Greenland Ice Sheet has been highlighted as a potentially important source of Fe, with elevated concentrations of highly reactive particulate Fe sourced from subglacial weathering (Bhatia et al., 2013; Hawkings et al., 2014, 2018) and high concentrations of Fe found in downstream fjords (Hopwood et al., 2016). Filterable Fe concentrations have also been studied for some mountain glacial systems, with concentrations and yields exceeding those from the Greenland Ice Sheet (Li et al., 2019; Schroth et al., 2011). However, Fe exports from glacial systems are highly variable, and there are still many critical unknowns regarding the factors controlling Fe production, the bioavailability of different phases, exchange between fjord and coastal waters, and how glacial nutrient exports are likely to change in the future (Hawkings et al., 2018; Hopwood et al., 2015; Li et al., 2019; Raiswell et al., 2018; Schroth et al., 2011, 2014).

Similar to Fe, silicon (Si) is a lithogenic element primarily sourced from chemical weathering of silicate bedrock and delivered to the ocean by rivers (Tréguer & De La Rocha, 2013; Tréguer et al., 1995). Si is an essential nutrient for siliceous organisms including diatoms, which account for up to 70% of primary productivity in the ocean (Nelson et al., 1995). Until recently, glaciers and ice sheets were not considered to be an important component of the Si cycle and were not included in global flux calculations (Frings et al., 2016; Tréguer & De La Rocha, 2013). Indeed, dissolved silicon (DSi) concentrations in glacier‐fed rivers tend to be very low, which has been interpreted to reflect limited silicate weathering rates due to near‐freezing temperatures at the base of glaciers (Anderson et al., 1997; M. Torres et al., 2017; Tranter et al., 2002). However, recent research has shown that glacial systems may actually provide large fluxes of a labile amorphous Si phase (ASi) bound to suspended sediment, which are believed to readily dissolve in seawater to produce bioavailable DSi (Blackburn et al., 2019; Hatton, Hendry, Hawkings, Wadham, Opfergelt, et al., 2019; Hawkings et al., 2017). By including the ASi component in addition to DSi, the total flux of bioavailable Si from Greenland has been approximated as 0.20 Tmol yr^−1^, equal to half the total DSi flux from all rivers that discharge into the Arctic Ocean (Dürr et al., 2011; Hawkings et al., 2017). This finding suggests that glacial meltwaters are an important source of Si to the ocean inventory and that increased glacial melt could support siliceous primary productivity. However, at present there are no published data related to ASi fluxes from glacial systems outside of the Greenland Ice Sheet or from catchments with variable glacial influence.

Chilean Patagonia represents a unique natural laboratory to investigate the environmental controls on riverine nutrient cycling. The region is characterized by very low levels of urbanization and agricultural development and the majority of rivers drain from near‐pristine catchments. Furthermore, Chilean Patagonia has a full spectrum of glacial influence, with rivers draining from completely deglaciated catchments, isolated mountain top glaciers, and the extensive glacial systems of the Patagonian ice fields. As such, this region represents an analog for progressive deglaciation, allowing for an investigation of how glacial cover influences Si and Fe exports. Glaciers in this region are currently experiencing the fastest global rates of glacial mass loss and retreat relative to their size (Dussaillant et al., 2019; Zemp et al., 2019), with the Patagonian ice fields shrinking by roughly 30 km^2^ per year from 1986–2011 (Davies & Glasser, 2012). North of the ice fields, in the Palena region of Patagonia, 374 mountain glaciers disappeared between 1985–2011 and over 100 new proglacial lakes formed (Paul & Mölg, 2014). Such dramatic and rapid landscape changes are likely to perturb physiochemical weathering budgets and biogeochemical cycles, affecting the export of freshwater, suspended particulate material (SPM), and nutrients to downstream ecosystems. Since Patagonia is a major source of terrigenous material to the South Pacific and the Southern Ocean, exports from this region likely play a critical role in regional productivity and biogeochemical cycling (Paparazzo et al., 2018). Currently, there are few published data on the geochemical composition of rivers from this region, despite the environmental and socioeconomic importance of the Patagonian fjords. This lack of data makes it challenging to predict how continued deglaciation will change terrestrial nutrient export and how this may impact downstream ecosystems in the future.

We present data from 40 river systems in Chilean Patagonia, with the objective to determine how glacial cover influences the concentrations and yields of Si and Fe. To do this, we report concentrations of dissolved, colloidal‐nanoparticulate and reactive particulate phases for both elements, exploring the dominant controls and influence of variable glacial cover on riverine concentrations. We use discharge data to calculate yield estimates (i.e., fluxes normalized by the upstream catchment area) of these nutrient phases to downstream ecosystems surrounding Patagonia. Finally, using the spectrum of glacial cover across this region, we apply a space‐for‐time substitution to predict how exports of bioavailable Si and Fe are likely to change in the future with continued glacial retreat.

## 2 Materials and Methods

### 2.1 Sampling Campaign

Water samples were taken from 40 rivers located between 42°S and 48°S along the Carretera Austral in Chilean Patagonia (Figure 2). The majority of samples were collected in January 2017, during the peak of the austral summer. Sampling locations were limited by accessibility and were chosen to match the locations of discharge gauging stations (Dirección General de Aguas). Water was sampled from fast‐flowing sections of the rivers and upstream of settlements. Aliquots for DSi were filtered immediately through 0.45 μm pore‐size Whatman GD/XP polypropylene (PP) syringe filters and stored in acid‐cleaned high‐density polyethene (HDPE) bottles. Fe samples were collected by sequentially filtering aliquots of river water through 0.45 μm Whatman GD/XP (PP) and 0.02 μm Whatman Anotop 25 syringe filters using trace‐metal clean protocols (Hawkings et al., 2014; Shiller, 2003). These pore sizes were chosen to differentiate between operationally defined colloidal‐nanoparticulate Fe (CNFe; 0.02–0.45 μm) and truly dissolved/soluble Fe (sFe, <0.02 μm), mirroring size fractions proposed by Shiller (2003) and used in multiple glacial‐river studies (Hawkings et al., 2014; Raiswell et al., 2018; Schroth et al., 2011). Particulate samples for SPM, ASi, and ascorbate‐extractable particulate Fe (FeA) analyses were collected from the majority of rivers by filtering 1–5 L of river water through pre‐weighed 47 mm diameter Millipore 0.45 μm polyethersulfone (PES) filters mounted onto an acid‐cleaned Nalgene filtration tower. SPM samples were only collected from near the surface of the river and so do not account for potential variability with depth(Bouchez et al., 2011). The exact volume of water that passed through the filter was recorded, and the filters were stored in the dark at 4°C until analysis.

### 2.2 Geospatial Analysis

**Figure 1:**
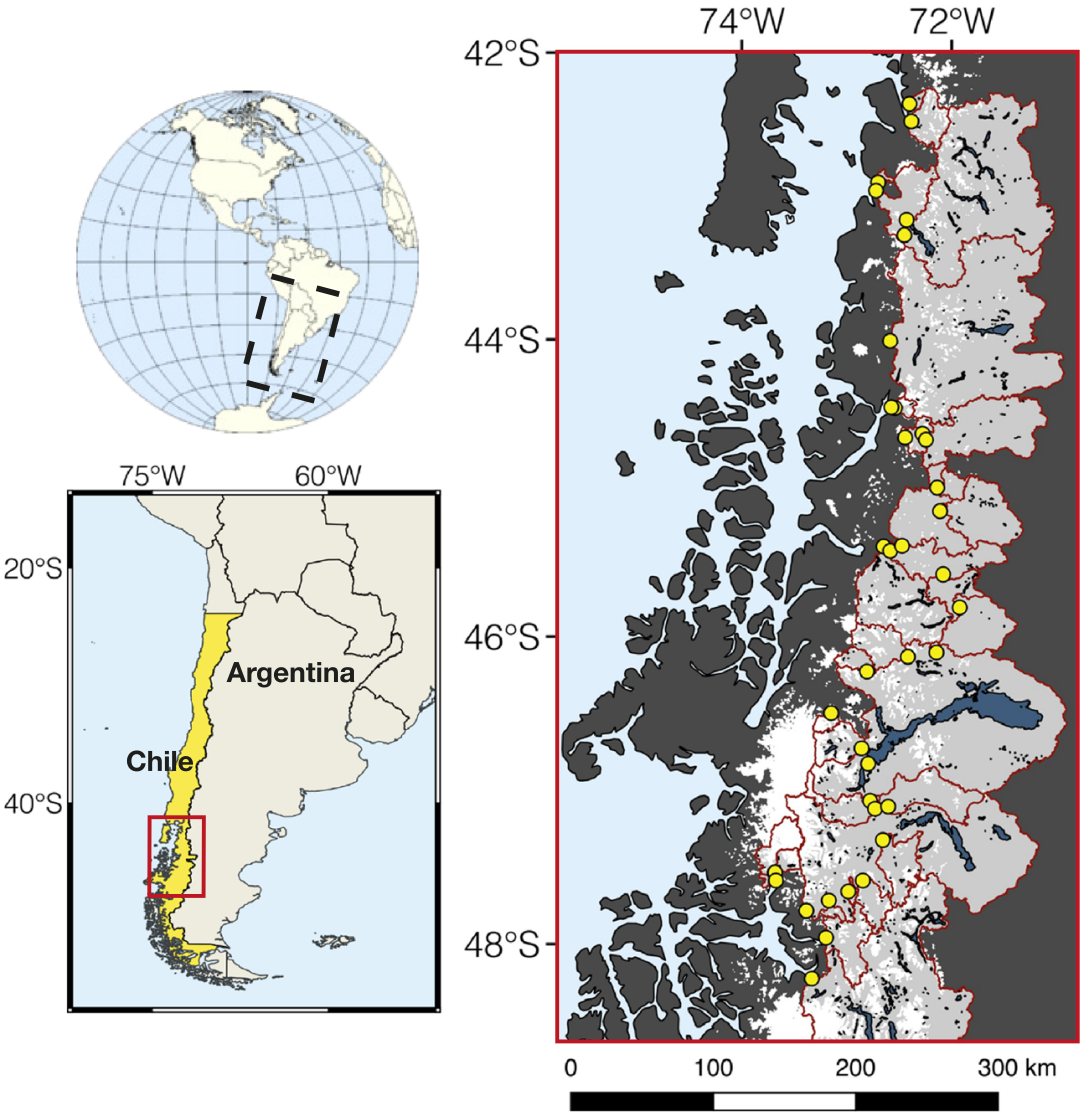
Map showing river sampling locations along the Carretera Austral in Chilean Patagonia. River sampling locations are shown by yellow dots and span catchments draining land from 42–48°S. Upstream catchment areas are shown in light gray. Catchment boundaries are shown in red and were modeled using Q‐GIS and Whitebox GAT. Large lakes are shown in blue and were taken from the Hydrolakes database (Messager et al., 2016). The glacial cover is shown in white and was taken from the Randolph Glaciers Inventory v6.0 (RGI Consortium, 2017).

Geospatial analyses were completed using a combination of Q‐GIS and Whitebox‐GAT (Lindsay, 2016). Digital elevation model (DEM) data of the region were sourced from the Shuttle Radar Topography Mission (SRTM) database (NASA), which gives elevation data at roughly 3‐arc sec (90 m) resolution. The DEM was used to delineate the upstream catchment area for all sampling locations by simulating the pathway of water and formation of channelized river networks across the topography. The modeled river networks accurately map the actual pattern of rivers across the region. Individual catchment areas range in size from 21.1–29,000 km^2^, with all river samples integrating a total upstream catchment area of ∼85,000 km^2^ (Figure 1). As this region is so close to the coast and most river sampling locations were less than 50 km from the river mouth, we speculate that geochemical concentration data will not significantly change further downstream as a result of in‐channel weathering, which has been shown to be minimal in proglacial rivers in Greenland (Urra et al., 2019).

Geospatial characteristics were calculated to quantify differences between catchments, allowing for a better understanding of how environmental parameters might influence the geochemistry of river systems. Glacial cover was calculated as a percentage of the total upstream catchment area, using outlines of glaciers sourced from the Randolph Glaciers Inventory database v6.0 (RGI Consortium, 2017). Relative glacial area calculations are not necessarily related to the contribution of glacial meltwater to total discharge at the time of sampling. However, there are insufficient data to provide any robust estimates of the proportion of meltwater from the sparse existing records of discharge and precipitation, given the catchments span such a large and climatically diverse region. As such, glacial cover was assigned as the best available and most reproducible proxy for comparing the relative influence of glaciers on different river systems across this region. The glacial cover of rivers sampled during this campaign ranged from 0–70% (supporting information Table S1). The glaciers in Chilean Patagonia are warm based, similar to other temperate regions (Hambrey & Glasser, 2012). Geospatial data sets for lithology (Hartmann & Moosdorf, 2012) and lake area (Messager et al., 2016) were also analyzed.

River catchments were classified into three categories based on their upstream glacial cover, to give broad insights into how Si and Fe budgets are likely to change with continued glacial retreat across Chilean Patagonia. These categories were <5% glacial cover (*n* = 16), where there was negligible glacial inputs, 5–20% glacial cover (*n* = 16) where there were moderate glacial inputs from tributary rivers, and >20% glacial cover (*n* = 8), where glacial meltwaters likely dominated total water flux and the main tributary was directly fed by a glacier. The significance between categories was measured using single‐factor analysis of variance (ANOVA) and unpaired, two‐tailed *t* tests assuming unequal variance.

### 2.3 Silicon Concentration Measurements

Dissolved Si (DSi) concentrations were measured in the LOWTEX facility at the University of Bristol using the LaChat 8500 Series‐2 flow injection analyzer (FIA) (QuikChem Method 31‐114‐27‐1‐D). DSi concentrations were measured colorimetrically, via the molybdenum blue complex to measure truly dissolved species in the form of silicic acid. Absorbance values were calibrated to standards of known Si concentration spanning the full range of sample Si concentrations. The mean limit of detection of this method was 0.7 μM, mean precision ±1.2%, and mean accuracy +2.6%, determined from replicates of a 250 ppb Si standard. We applied an error of ±2.5% to all DSi measurements, representing ±2*σ* variability of replicate samples and standards that span the full range of river concentrations.

Colloidal‐nanoparticulate size Si (CNSi) concentrations were measured by refiltering aliquots of 0.45 μm GD/XP filtered sample through 0.02 μm Whatman Anotop 25 filters. Samples were then passed through cation exchange columns using the method developed by Georg et al. (2006) to remove matrix interferences and isolate Si. The signal intensity of 0.45 and 0.02 μm filtered aliquots was measured by multi‐collector inductively coupled plasma mass spectrometry (MC‐ICP‐MS) at the University of Bristol and compared to standards of known concentration to give Si concentrations for each size fraction. Unlike the FIA, which only measures truly dissolved Si in the form of silicic acid, the MC‐ICP‐MS ionizes all Si present. CNSi was quantified as the difference in concentration between the <0.45 and <0.02 μm filtered aliquots for each river sample. A conservative error of ±5% was assigned, representing ±2*σ* variability of replicate samples.

Concentrations of reactive amorphous Si (ASi) associated with SPM were measured using an alkaline extraction method developed by DeMaster (1981). This method is commonly used to measure ASi and has been used by several recent publications investigating glacial river sediments (Hatton, Hendry, Hawkings, Wadham, Kohler, et al., 2019; Hatton, Hendry, Hawkings, Wadham, Opfergelt, et al., 2019; Hawkings et al., 2017). Approximately 30 mg of SPM was precisely weighed and combined with 50 ml of 0.1 M Na_2_CO_3_ solution. A temperature of 85°C was maintained throughout the extraction, and 1 ml aliquots were taken at 2, 3, and 5 hr intervals before analysis via FIA, as described above. These data were multiplied by the dilution factor of the extractant and divided by the weight of sediment added to each sample to give Si concentrations as a percentage of total dry sediment weight (wt.%) for each time point. ASi concentrations for each sample were determined by calculating the *y* intercept of the linear regression for the three time points for each sample. This method assumes that ASi phases dissolve rapidly within the first hour and that clays and other more refractory materials release Si at a linear rate throughout the extraction. A conservative error of this method was calculated as ±10%, representing ±2*σ* variability of replicate samples spanning the full range of river concentrations. ASi values were also reported in μM, derived by converting from wt.% to μM g^−1^ of ASi and then multiplying by the SPM concentration of the river, with a propagated error of ±7.5%.

### 2.4 Iron Concentration Measurements

Truly dissolved/soluble Fe (sFe; <0.02 μm) and <0.45 μm filterable Fe were measured using a Thermo Scientific X‐SERIES 2 quadrupole ICP‐MS at the National Oceanography Centre, Southampton. Beryllium, indium and rhenium were used as internal standards to correct for drift and matrix effects. External calibration solutions were made to match the concentration range observed in samples. Precision was ±2.3% for Fe concentrations above 10 nM and ±15.3% for concentrations below 10 nM to the limit of detection (∼3 nM). Accuracy was always within 10% of a gravimetrically weighed check standard (161 nM). Colloidal‐nanoparticulate Fe (CNFe) concentrations were calculated as the difference between <0.02 and <0.45 μm filterable Fe.

Ascorbate extractable particulate iron (FeA; >0.45 μm) concentrations were analyzed using the ascorbate leach (Raiswell et al., 2010). This method is calibrated to extract mostly highly reactive two‐line ferrihydrite and surface‐bound Fe(II) (Raiswell et al., 2018), which have been shown to be at least partially bioavailable (Nodwell & Price, 2001; Wells et al., 1983). SPM samples were extracted for 24 hr using an ascorbic acid solution buffered to pH 7.5 and analyzed with a Thermo Scientific Gallery discrete analyzer using the ferrozine colorimetric protocol (Viollier et al., 2000). Standards were matrix‐matched and spanned the concentration range observed in samples. Concentrations of FeA are reported as a percentage of the dry sediment weight (wt.%) with a conservative error of ±10% (±2*σ*) and in μM with a propagated error of ±7.5%.

### 2.5 Composition of Colloidal‐Nanoparticulate Material

Colloidal‐nanoparticulate size material was removed from solution 18 months after collection, by refiltering a <0.45 μm aliquot onto 0.22 μm cellulose‐nitrate membrane filter using acid‐cleaned syringes and Swinnex filter holders. The Si concentration of the <0.22 μm filtered aliquot was also tested with MC‐ICP‐MS, giving assurance that filtering through this pore size removed the majority of CNSi. The DSi concentrations were also measured 18 months after collection to ensure that there had been no dissolution or precipitation of CNSi between collection and when the colloidal particles were removed from the solution. The composition of the colloidal particles was only tested for one sampling location (PFC01‐SA; Río Huemules) due to limited sample volumes for the other sites. However, the geology underlying the majority of glacier‐fed river sites is similar (Patagonian granite batholith; Hervé et al., 2007; Pankhurst et al., 1999), and so we assume the that composition is broadly representative of the colloidal‐nanoparticulate material across the region.

The colloidal‐nanoparticulate material was imaged using a Hitachi S‐3500N scanning electron microscope (SEM), and estimates of elemental ratios were measured using a ThermoNoran energy dispersive X‐ray spectrometer (EDX) at the University of Bristol. The composition of the colloidal phase was also investigated using a ThermoNicolet i10 Fourier‐transform infrared (FTIR) spectrometer fitted with a Ge‐tipped Attenuated Total Reflectance (ATR) head. Scans (128–256) were collected from 650–4,000 cm^−1^ at a resolution of 4 cm^−1^ using a MCT detector and a KBr beam splitter in absorbance mode. The effective collection area was approximately 50–30 μm^2^, and a spectrum of the filter ensured that this was not present in the sample data. The potential lability of CNSi and CNFe was also tested using a 0.1 M Na_2_CO_3_ alkaline extraction and an ascorbate extraction respectively, using similar protocols as outlined in sections 2.3 and 2.4. Full details of the colloidal‐nanoparticulate extractions are given in the supporting information.

### 2.6 Si and Fe Yield Calculations

Estimates of the yields (fluxes normalized by catchment area) of dissolved, colloidal‐nanoparticulate and reactive SPM‐bound Si and Fe were calculated for all catchments where discharge data were available (*n* = 23). Most discharge data were sourced from the Dirección General de Aguas database unless specified otherwise (supporting information Table S2). Mean monthly discharge data were compiled, and a mean annual discharge value was calculated from all years with complete records. No discharge data were available from some of the smaller catchments, but the majority of large catchments have reliable records. Samples were collected close to the discharge monitoring stations, and so values directly corresponded to our sampling locations. Mean annual yields were estimated by multiplying riverine nutrient concentrations by the mean annual discharge, before normalizing to the upstream catchment area to give yields in units of megagrams (Mg) km^−2^ yr^−1^. These data represent broad estimates as mean annual discharge values were used, which can oversimplify hydrological budgets, and data do not account for temporal or seasonal variability in riverine concentrations.

## 3 Results

### 3.1 SPM Concentrations

SPM concentrations from rivers in Chilean Patagonia ranged from 0.5–240.8 mg L^−1^ (mean = 29.9 ± 45.5 mg L^−1^ (±1*σ*), *n* = 36). The river systems with the highest SPM concentrations drained from active volcanoes, large glacial systems, and catchments with pyroclastic and sedimentary lithologies. Rivers with the lowest SPM concentrations were located at the outlets of large lake systems or from small catchments draining metamorphosed bedrock. Glacier‐fed rivers (i.e., >20% glacial cover) had a mean SPM concentrations of 62.0 ± 26.5 mg L^−1^ (range = 22.4–87.5 mg L^−1^, *n* = 6), roughly 4 times the mean for non‐glacial and non‐volcanic rivers in this region (mean = 15.6 ± 21.5 mg L^−1^; *t*_6_ = 4.0, *p* < 0.01).

### 3.2 Riverine Silicon Concentrations

**Figure 2:**
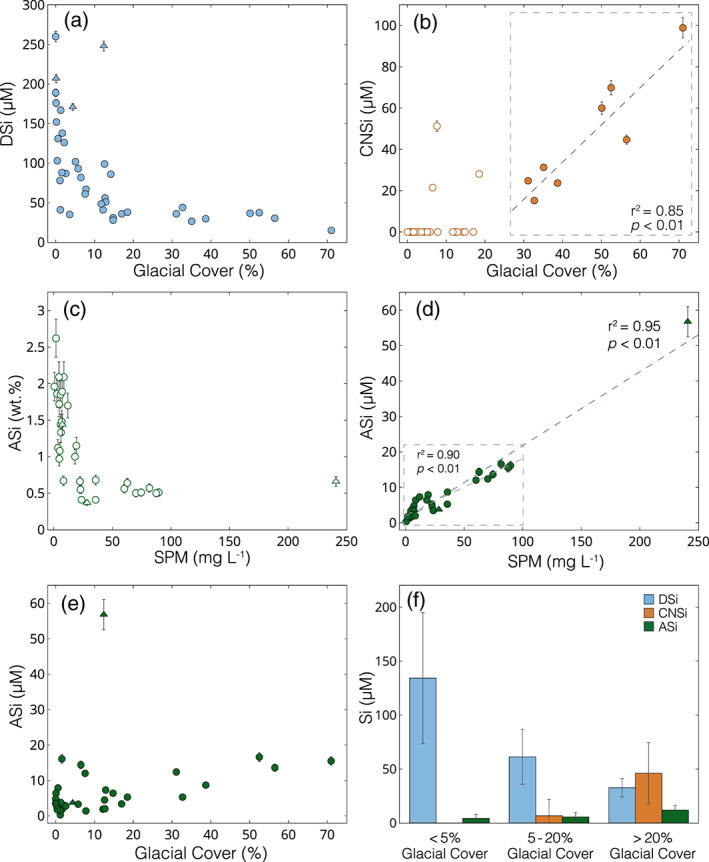
Riverine silicon concentration data. Rivers draining from active volcanoes are shown with a triangle and all other river systems are shown with a circle. (a) Dissolved Si (silicic acid; DSi) (μM) versus glacial cover (%). (b) Colloidal‐nanoparticulate Si (0.02–0.45 μm; CNSi) (μM) versus glacial cover (%)—unfilled circles excluded from linear regression. (c) Amorphous Si (>0.45 μm; ASi) (wt.%) versus suspended particulate material (SPM) load (mg L^−1^). (d) ASi (μM) versus SPM load (mg L^−1^). (e) ASi (μM) versus glacial cover (%). (f) Bar plots showing mean ±1*σ* DSi, CNSi, and ASi concentrations for catchments categorized by glacial cover (%).

Concentrations of riverine dissolved Si (DSi; as silicic acid) ranged from 15.5–259.6 μM (mean = 89.3 ± 64.2 μM, *n* = 40) and showed an inverse logarithmic relationship with glacial cover (*r* ^2^ = 0.58; Figure 2a). Rivers with >20% glacial cover had low DSi concentrations, with values from 15.5–43.8 μM (mean = 32.0 ± 8.6 μM, *n* = 8). River systems with less or no glacial influence (<20% glacial cover) had broad range of DSi concentrations between 30.9 and 259.6 μM (mean = 103.6 ± 64.1 μM, *n* = 32).

A significant colloidal‐nanoparticulate size Si component (CNSi; 0.02–0.45 μm) was found in all glacially fed river systems. CNSi concentrations ranged from 15.2–98.9 μM (mean = 46.1 ± 28.5 μM, *n* = 8) and directly scaled with glacial cover for glacier‐fed river systems with >20% glacial cover (r^2^ = 0.85, *p* < 0.01; Figure 2b). CNSi was also detected in three catchments with <20% glacial cover, with concentrations of CNSi from 21.5–51.3 μM. These catchments receive significant meltwater inputs from the northern and southern Patagonian ice fields but represent areas where the percentage glacial cover was disproportionately low due to large catchment sizes (>15,000 km^2^). No measurable quantities of CNSi were observed in any other river systems, with the Si concentration of <0.45 μm filtered samples within analytical error of the DSi. CNSi dominated the total Si budget of most glacier‐fed rivers, with mean CNSi concentrations 1.4 times higher than mean DSi and 3.8 times higher than mean ASi. The concentration of CNSi did not vary as a function of glacier size and was present in rivers fed by both large outlet glacial systems and isolated mountain‐top glaciers with areas of just ∼5 km^2^. The presence of CNSi phases also did not depend on the underlying bedrock type, with CNSi phases found in systems draining from granite bedrock of the Patagonian granite batholith, basic igneous lithologies, and metamorphosed basement rocks (Hartmann & Moosdorf, 2012; supporting information Table S1).

Concentrations of amorphous Si (ASi; >0.45 μm) ranged from 0.37–2.62 wt.% (mean = 1.12 ± 0.63 wt.%, *n* = 33; Figure 2c). No trend was observed between ASi wt.% values and the DSi concentration of the river water (*r* ^2^ = 0.14; *p* > 0.05). Glacier‐fed rivers showed a narrow range of ASi, with values from 0.50–0.68 wt.% (mean = 0.57 ± 0.08 wt.%). An inverse logarithmic relationship was observed between the SPM load and ASi (wt.%) concentrations (*r* ^2^ = 0.61; Figure 2c). All rivers with high SPM loads (>20 mg L^−1^) had low ASi wt.% (<0.7 wt.%) and those with lower SPM concentrations (<20 mg L^−1^) had high ASi values (>1 wt.%; *t*_20_ = 8.7, *p* < 0.01). When expressed as absolute concentrations, ASi ranged from 0.32–56.8 μM (mean = 7.73 ± 10.0 μM) and were almost entirely controlled by the SPM concentration (*r* ^2^ = 0.95; *p* < 0.01; Figure 2e).

Systematic differences between catchments were revealed when categorized by glacial cover (Figure 2f and supporting information Table S3). First, DSi concentrations decreased with increasing glacial cover, with mean DSi concentrations roughly 4 times higher for catchments with <5% glacial cover, compared to mean values for catchments with >20% glacial cover (*t*_16_ = 6.5, *p* < 0.01) and roughly twice as high as for catchments with 5–20% glacial cover (*t*_20_ = 4.4, *p* < 0.01). Mean CNSi values from catchments with >20% were 46.1 ± 28.5 μM and accounted for the largest component, roughly equaling the total concentration of DSi and ASi. Mean ASi concentrations from catchments with >20% glacial cover were more than double catchments <20% glacial cover, with mean concentrations of 12.0 ± 4.3 μM, compared to 4.9 ± 3.9 μM (*t*_7_ = 3.7, *p* < 0.01). ASi concentrations were not significantly different between catchments with <5% glacial cover or 5–20% glacial cover (*t*_20_ = 0.8, *p* > 0.05; supporting information Table S3).

### 3.3 Riverine Iron Concentrations

**Figure 3:**
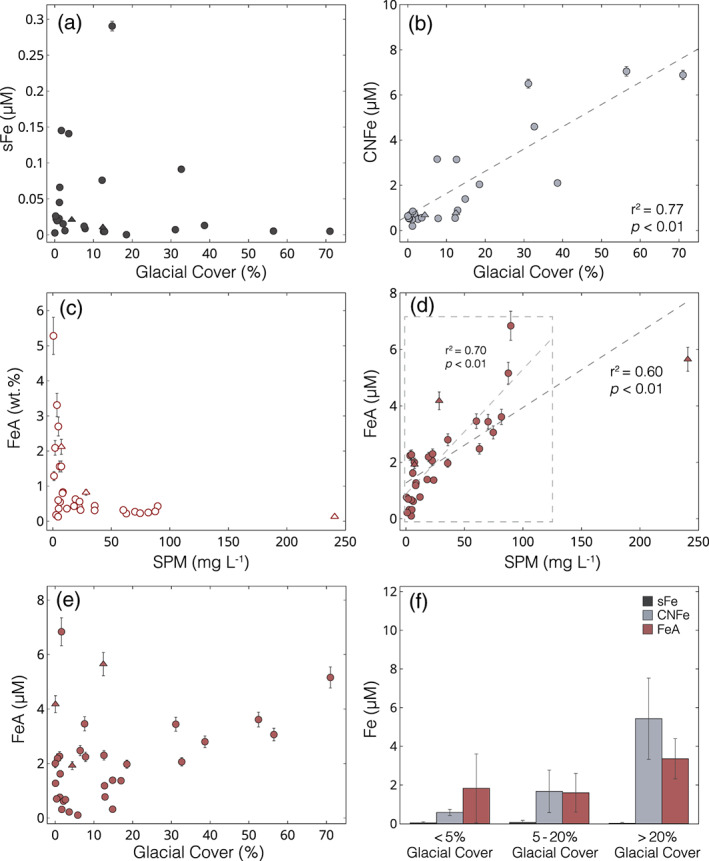
Riverine iron concentration data. Rivers draining from active volcanoes are shown with a triangle, and all other river systems are shown with a circle. (a) Soluble Fe (<0.02 μm; sFe) (μM) versus glacial cover (%).(b) Colloidal‐nanoparticulate Fe (0.02–0.45 μm); CNFe) (μM) versus glacial cover (%); (c) Ascorbate‐extractable Fe (>0.45 μm; FeA) (wt.%) versus SPM load (mg L^−1^). (d) FeA (μM) versus SPM load (mg L^−1^). (e) FeA (μM) versus glacial cover (%). (f) Bar plots showing mean ±1σ sFe, CNFe, and FeA concentrations for catchments categorized by glacial cover (%).

Concentrations of truly dissolved/soluble Fe (sFe; <0.02 μm) ranged from below detection (<3 nM) to 290 nM (mean = 44.2 ± 66.7 nM, *n* = 24) and showed no relationship with glacial cover (*r* ^2^ = 0.02, *p* > 0.05; Figure 3a). Rivers with sFe concentrations exceeding 100 nM (*n* = 3) were organic‐rich, brown water river systems with inputs from peatbogs or wetlands. Glacier‐fed rivers have mean sFe concentrations of 24.3 ± 37.6 nM (*n* = 5), roughly half the mean for other river systems in this region (mean = 49.4 ± 72.4 nM, *n* = 19), although the groups were not statistically different (*t*_13_ = 1.1, *p* > 0.05). Colloidal‐nanoparticulate Fe (CNFe; 0.02–0.45 μm) concentrations ranged from 0.19–7.05 μM (mean = 1.85 ± 2.10 μM, *n* = 26) and increased linearly with increasing glacial cover (*r* ^2^ = 0.77, *p* < 0.01; Figure 3b). The highest CNFe concentrations were from glacially fed rivers, with values ranging from 2.10–7.05 μM (mean = 5.43 ± 2.10 μM, *n* = 5); more than 5 times higher than the mean for other river systems in this region (mean = 1.00 ± 0.83 μM, *n* = 21; *t*_4_ = 4.7, *p* < 0.01).

Concentrations of ascorbate‐extractable Fe (FeA; >0.45 μm) ranged from 0.13–5.28 wt.% (mean = 0.93 ± 1.12 wt.%, *n* = 32; Figure 3c). Glacier‐fed rivers showed a narrow range of FeA concentrations, with values from 0.23–0.44 wt.% (mean = 0.31 ± 0.09 wt.%, *n* = 6). All rivers with high SPM loads (>50 mg L^−1^) had FeA concentrations below 0.5 wt.%. Conversely, all river systems with FeA values >1 wt.% had SPM concentrations <10 mg L^−1^, similar to the trend observed between SPM and ASi (wt.%) values (Figure 2c). When normalized to the SPM load, FeA (μM) concentrations ranged from 0.10–6.84 μM (mean = 2.15 ± 1.63 μM, *n* = 32). The SPM concentration of rivers correlated with FeA (μM) concentrations (*r* ^2^ = 0.60; *p* < 0.01; Figure 3d), although with more variability than ASi. The highest FeA (μM) concentrations were from non‐glacial, high SPM load rivers draining from volcanoes or sedimentary bedrock. Glacier‐fed rivers had also high FeA concentrations ranging from 2.06–5.16 μM (mean = 3.36 ± 1.04 μM, *n* = 6; Figure 3e).

When grouped by glacial cover, sFe concentrations were not significantly different between categories (*F*_2_ = 0.3, *p* > 0.7; Figure 3f). CNFe concentrations increased steeply with increasing glacial cover, with mean values an order of magnitude higher for catchments with >20% glacial cover compared to <5% glacial cover (*t*_4_ = 5.1, *p* < 0.01). Concentrations of FeA also increased with greater glacial cover, with mean values roughly twice as high for catchments with >20% glacial cover compared to <5% glacial cover (*t*_16_ = 2.4, *p* < 0.05) or 5–20% glacial cover (*t*_10_ = 3.4, *p* < 0.01; supporting information Table S4).

### 3.4 Colloidal‐Nanoparticulate Composition

**Figure 4:**
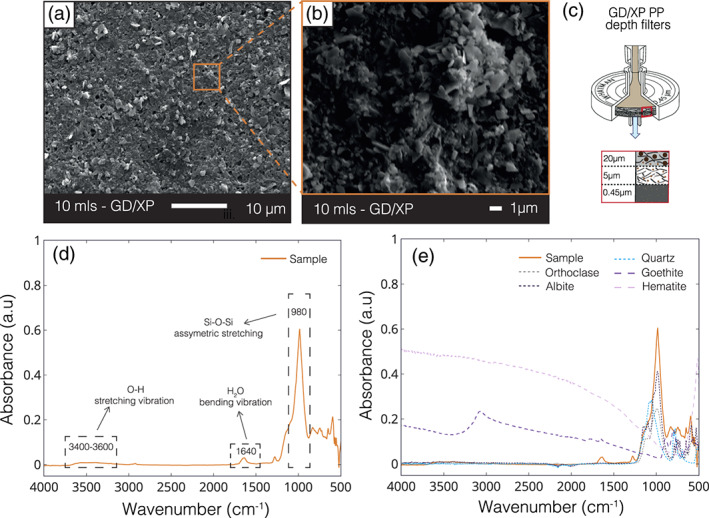
SEM images and FTIR‐ATR absorbance spectra of colloidal‐nanoparticulate material. (a and b) SEM images of 10 ml of Río Huemules (PFC01‐SA; Río Huemules—70% glacial cover) filtered river water sample. Samples were initially filtered through 0.45 μm GD/XP depth filter capsules, as shown in schematic (c). The colloidal‐nanoparticulate material was then removed from solution by filtering 10 ml of sample through a 0.22 μm cellulose nitrate sheet filter. (d) Line graph showing absorbance spectrum versus wavenumber (cm^−1^) of glacier‐fed river colloidal‐nanoparticulate material (orange line). The colloidal‐nanoparticulate material IR spectra are missing the narrow, sharp, OH peaks that would be indicative of clays or sheet silicate weathering products. (e) Colloidal‐nanoparticulate material composition compared to various silicate minerals, using data sourced from the RRUFF database (dotted/dashed lines), with spectra most similar to albite feldspar (Lafuente et al., 2015).

SEM images of the glacial particles revealed the abundance of colloidal‐nanoparticulate material, with just 10 ml of a refiltered glacier‐fed river sample completely covering the filter membrane (Figures 4a and 4b). FTIR analysis provided evidence that the colloidal‐nanoparticulates were mainly composed of primary aluminosilicate minerals, with absorbance spectra most similar to albite feldspars (Figures 4d and 4e). However, the spectra were complex, and other phases may also be present. SEM‐EDX analyses indicated that the bulk composition of the colloids was similar to alkali feldspars, although with elevated Mg (∼1.6 wt.%) and Fe (∼4.7 wt.%), suggesting additional Mg‐ or Fe‐rich phases such as biotite or hornblende minerals from the granite or adsorbed Fe‐oxyhydroxide phases (supporting information Table S5). Repeat measurements of DSi concentrations of river waters, 1–18 months after collection were within analytical error, indicating that the filtered samples were stable during storage with no further dissolution or precipitation of CNSi within this time period. Si concentrations of the <0.22 μm filtered size fraction were within error of the DSi concentrations, showing that >90% of CNSi phases were removed with this pore size, likely due to significant flocculation during storage. An alkaline extraction on the colloidal‐nanoparticulate material gave evidence that <5% of the CNSi was ASi. Extraction on the colloidal‐nanoparticles indicated that ∼15% of the CNFe was ascorbate‐extractable iron (FeA). However, the CNFe samples had been stored for ∼2.5 years in solution before extraction, and so aging effects of ferrihydrite were likely to be significant (Raiswell et al., 2018).

### 3.5 Yield Estimates

**Figure 5:**
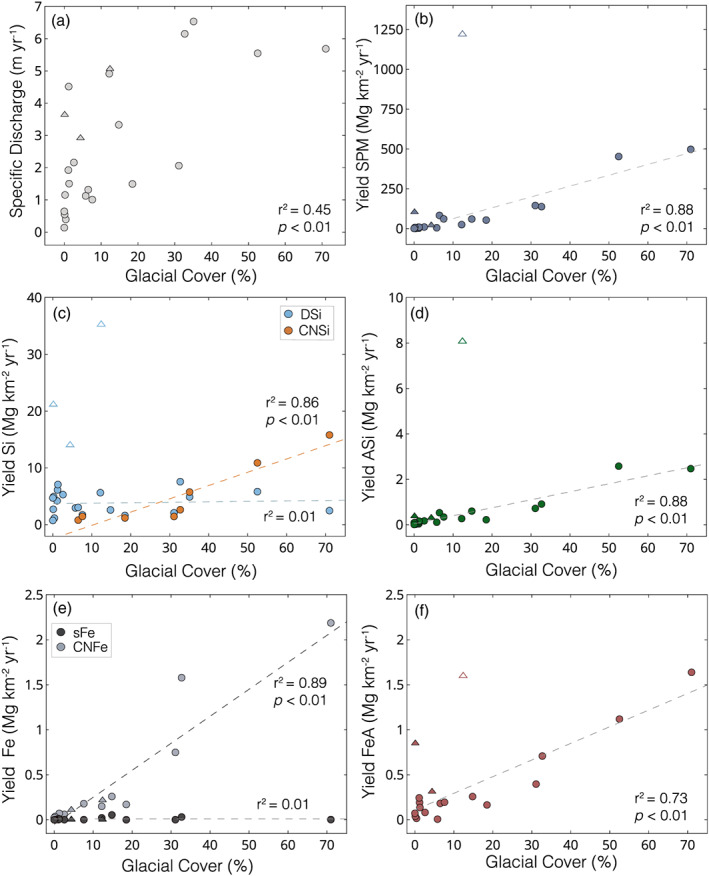
Si and Fe yield estimates. (a) Specific discharge (m yr^−1^) versus glacial cover (%). (b) Yield SPM (Mg km^−2^ yr^−1^) versus glacial cover. (c) Yield DSi and CNSi versus glacial cover. (d) Yield ASi versus glacial cover.(e) Yield sFe and CNFe versus glacial cover. (f) Yield FeA versus glacial cover. Rivers draining from active volcanoes are shown with a triangle, and all other river systems are shown with a circle. Unfilled symbols are excluded from linear regression calculations.

Specific discharge for the catchments ranged from 0.14–6.54 m yr^−1^ (mean = 2.77 ± 2.06 m yr^−1^, *n* = 23) and showed a positive correlation with glacial cover, but with considerable variability from other factors (*r* ^2^ = 0.45, *p* < 0.01; Figure 5a). Yields (i.e., fluxes normalized by catchment area) of SPM ranged from 0.75–1,221 Mg km^−2^ yr^−1^ (mean = 138.5 ± 283.6 Mg km^−2^ yr^−1^, *n* = 21; Figure 5b). Excluding one volcanic catchment (PTR01‐R23; Río Ibanez) that had extremely high SPM yields, our data showed a strong linear trend of increasing SPM yields with increasing glacial cover (*r* ^2^ = 0.88, *p* < 0.01). Yields of DSi ranged from 0.74–35.3 Mg km^−2^ yr^−1^ (mean = 6.39 ± 7.74 Mg km^−2^ yr^−1^, *n* = 23; Figure 5c) and were highest in catchments draining from the two active volcanoes in our sampling region. Excluding the volcano‐fed systems, all other catchments had DSi yields below 8 Mg km^−2^ yr^−1^ and showed no trend with glacial cover (*r* ^2^ = 0.00, *p* > 0.05). In rivers where CNSi was present, yields ranged from 0.80–15.8 Mg km^−2^ yr^−1^ (mean = 4.99 ± 5.54 Mg km^−2^ yr^−1^, *n* = 8) and showed a significant linear relationship with glacial cover (*r* ^2^ = 0.86, *p* < 0.01; Figure 5c). Yields of ASi ranged from 0.01–8.08 Mg km^−2^ yr^−1^ (mean = 0.83 ± 1.76 Mg km^−2^ yr^−1^, *n* = 22) and values directly scaled with the yield of SPM (*r* ^2^ = 0.99, *p* < 0.01) and glacial cover (*r* ^2^ = 0.88, *p* < 0.01; Figure 5d).

Yields of sFe were all below 0.05 Mg km^−2^ yr^−1^ (mean = 0.009 ± 0.015 Mg km^−2^ yr^−1^, *n* = 16) and showed no relationship with glacial cover (*r* ^2^ = 0.01, *p* > 0.05; Figure 5e). Yields of CNFe ranged from 0.01–2.19 Mg km^−2^ yr^−1^ (mean = 0.37 ± 0.63 Mg km^−2^ yr^−1^, *n* = 16; Figure 5e) and showed a significant positive linear relationship with glacial cover (*r* ^2^ = 0.89, *p* < 0.01). Yields of FeA ranged from 0.01–1.64 Mg km^−2^ yr^−1^ (mean = 0.43 ± 0.51 Mg km^−2^ yr^−1^, *n* = 19) and were highest from glaciated and volcanic catchments (Figure 5f). Excluding a volcano‐fed catchment with high SPM loads, yields of FeA showed a positive correlation to glacial cover (*r* ^2^ = 0.73, *p* < 0.01), similar to the trend for ASi and SPM. The data used for yield estimates are summarized in supporting information Tables S1 and S2.

## 4 Discussion

### 4.1 Controls on Riverine Si Concentrations and Composition

The low concentrations of DSi found in the glacier‐fed rivers of Patagonia are consistent with concentrations observed in proglacial rivers worldwide (Anderson, 2007; M. Torres et al., 2017). Such low and relatively invariant DSi concentrations have been thought to reflect limited silicate mineral weathering in the low‐temperature conditions beneath glaciers, combined with dilution by supraglacial meltwaters (Anderson et al., 1997; M. Torres et al., 2017; Tranter et al., 2002). By contrast, the wide range of DSi concentrations in non‐glacial rivers in Patagonia highlights the range of complex processes that can affect Si cycling across the region. The lowest non‐glacial riverine DSi concentrations drain from small catchments dominated by metamorphosed carbonate or mafic bedrock (supporting information Table S1), which contain low proportions of Si and are resistant to weathering respectively. The highest DSi concentrations are all in catchments draining or proximal to active volcanoes, likely due to the exposure of highly reactive, fine‐grained volcanic regolith that is rich in Si.

In a previous study of DSi in Patagonian rivers, the variability of DSi concentrations was attributed to the thickness of volcanic ash soils (andosols) in the upstream catchment area (Vandekerkhove et al., 2016, *n* = 5). Our data from the same rivers replicate the previous correlation, supporting this interpretation (*r* ^2^ = 0.98, *p* < 0.05, *n* = 4; supporting information Figure S1). However, when we include the additional rivers sampled during this study, the relationship weakens (*r* ^2^ = 0.41, *p* < 0.01, *n* = 36; supporting information Figure S1). While some of the highest DSi concentrations are from catchments with high mean andosol thickness, our data set demonstrates that other factors such as bedrock, soil and vegetation types, catchment area, topography, climate, and the presence of lakes likely play important roles as well. Without local‐scale experiments and detailed observations of temporal variability, it is not possible to deconvolve all of the processes affecting riverine DSi concentrations in Patagonia. However, it is clear that glacial cover, volcanic debris, and rock type play first‐order roles in setting DSi concentrations, which may later be modified by additional processes such as secondary weathering or biological productivity.

Colloidal‐nanoparticulate size Si (0.02–0.45 μm; CNSi) dominated the Si budget in all glacier‐fed river systems in Patagonia, with concentrations up to 7 times higher than the respective DSi concentration (Figure 2b and supporting information Table S1). As the CNSi phase was found in all glacier‐fed rivers, irrespective of glacier size, we assume that this phase is a subglacial weathering product, and its formation does not depend on glacial size and associated subglacial water residence times. Indeed, compositional analysis of the colloidal‐nanoparticulate phase demonstrates that they are mainly composed of finely ground alkali feldspars (Figures 4d and 4e), indicating that this phase is formed primarily by comminution of the bedrock rather than by chemical weathering. We assume that other major components of the granite bedrock such as quartz are not found in the colloidal‐nanoparticulate size fraction due to higher resistance to physical weathering. Our results suggesting that <5% of the CNSi was Na_2_CO_3_ extractable indicate that ASi phases are negligible within this size fraction and that the CNSi may dissolve less readily than ASi in seawater. Feldspar minerals are traditionally considered to have low solubility in the ocean, although recent research has suggested that the dissolution of feldspars in seawater may be more significant than previously appreciated and should be accounted for to resolve marine elemental budgets (Gruber et al., 2019; Jeandel & Oelkers, 2015). Furthermore, rock‐crushing experiments have also demonstrated that submicron‐size feldspar particles are formed during physical grinding processes and that these phases are highly unstable in solution and rapidly dissolve to form DSi (Holdren & Berner, 1979). These findings suggest that the CNSi feldspar phases identified in glacial rivers in Patagonia may have some degree of lability in seawater, especially as their submicron size may prolong their time in the water column before settling, enabling further weathering and offshore transport. The possibility of a sizeable undocumented pool of potentially labile Si being exported from glacier‐fed rivers has clear implications for resolving biogeochemical cycles, with further research needed to quantify the solubility, transformations and fate of CNSi phases within downstream ecosystems.

High concentrations of CNSi have also been reported from proglacial rivers in Alaska (mean = 61.2 ± 67.6 μM) and were absent from rivers draining boreal forested catchments (Schroth et al., 2011), reinforcing interpretations of a subglacial origin. From analyses of the SPM fraction, CNSi phases in Alaska were inferred to be Fe‐bearing silicates such as amphibole, biotite, smectite, and chlorite (Schroth et al., 2011). The similar concentrations of CNSi in Patagonia and Alaska suggest that these phases may be a common feature of proglacial rivers, although with compositional variability related to the underlying bedrock. However, few other studies have investigated CNSi in rivers, and it is currently unclear whether these phases were absent or potentially overlooked due to sampling or analytical protocols. Several methods are routinely used to quantify DSi concentrations, including colorimetric techniques, which only detect chemically dissolved species (e.g., silicic acid) and ICP methods, which ionize and detect all phases including colloidal‐nanoparticulate species. As such, CNSi phases could go undetected by colorimetric methods, or the DSi components could be significantly overestimated if <0.45 μm filtered fractions are measured by ICP, and CNSi phases are present. Currently, it is not known whether CNSi phases are ubiquitous in glacier‐fed rivers, whether their formation and abundance depend on the underlying bedrock type or subglacial conditions, how their composition could vary between regions, and how this might affect potential lability in downstream ecosystems.

In addition to filterable Si phases that pass through a 0.45 μm filter (DSi + CNSi), recent studies have shown that rivers can also transport substantial quantities of amorphous Si (ASi), which are solid non‐crystalline Si phases associated with the SPM (i.e., >0.45 μm) load (Conley, 1997; Frings et al., 2014; Hawkings et al., 2017; Tréguer & De La Rocha, 2013). The Na_2_CO_3_ extraction protocols are used to capture the highly reactive ASi component, which has been shown to readily dissolve in seawater to form bioavailable DSi (Frings, 2017; Frings et al., 2014; Hawkings et al., 2017). ASi can be both biogenic (BSi), such as diatoms, radiolaria, and plant phytoliths, or abiogenic, such as volcanic glass or secondary weathering and pedogenic products (Barão et al., 2014; Frings et al., 2014). The magnitude of riverine ASi export is poorly constrained, despite evidence that fluxes of ASi and DSi could be of similar magnitude (Frings et al., 2014). Indeed, reactive ASi phases were found in all river systems sampled in Chilean Patagonia and represent a key component of Si budgets in this region. The high ASi (wt.%) values in Patagonia (1.00–2.62 wt.%) were all in non‐glacial rivers draining from large lakes or through catchments with grasslands, likely reflecting the inclusion of biogenic silica phases, such as diatoms and plant phytoliths. The low ASi wt.% values (0.41–0.68 wt.%) were all from high SPM systems, likely due to inhibited diatom productivity in turbid river waters and dilution by primary aluminosilicate minerals. The range of ASi (wt.%) values from glacial rivers in Patagonia (0.50–0.68 wt.%) were comparable to data from glacial systems in Greenland (0.23–0.91 wt.%; Hatton, Hendry, Hawkings, Wadham, Kohler, et al., 2019; Hawkings et al., 2017) and Alaska (0.14–0.64 wt.%; Hatton, Hendry, Hawkings, Wadham, Opfergelt, et al., 2019), suggesting similarities in composition and formation mechanisms, despite differences in bedrock, glacier size and subglacial residence times.

Given that the ASi (wt.%) values have a relatively narrow range between different river systems, ASi (μM) concentrations are primarily controlled by the SPM load (Figure 2d), similar to the relationship reported by Frings et al. (2014) from the Ganges. As such, the highest ASi (μm) concentrations in our data set are all from rivers with high SPM loads such as catchments draining from volcanoes, where there is highly erodible regolith, or from glaciers, where there is active comminution of bedrock by moving ice. In highly glaciated catchments, the ASi component can equal the DSi, highlighting that these phases need to be accounted for in glacial nutrient exports. At present, there is considerable uncertainty about the formation mechanism of ASi beneath glaciers, with an ongoing debate about whether ASi is formed purely by mechanical grinding processes or if chemical weathering reactions could also play a role (Blackburn et al., 2019; Hatton, Hendry, Hawkings, Wadham, Opfergelt, et al., 2019; Hawkings et al., 2017).

The majority of subglacial weathering budgets are based purely on the dissolved major ion ratios of glacial outflow, with enhanced sulfate and bicarbonate ion concentrations indicating that the dominant weathering pathways beneath glaciers are carbonate dissolution and sulfide oxidation (M. Torres et al., 2017; Tranter et al., 2002; Urra et al., 2019). These combined weathering reactions ultimately release CO_2_ to the atmosphere, leading to the idea that glacial weathering processes could help to stabilize net cooling feedbacks during glacial periods (Sharp et al., 1995; M. Torres et al., 2017). However, most studies have been limited to smaller mountain glaciers, which appear to display different weathering pathways compared with larger ice sheet catchments (Michaud et al., 2016; Wadham et al., 2010; Urra et al., 2019). Furthermore, if glaciogenic ASi phases are a product of chemical weathering, their formation and abundance could mean that silicate weathering rates, the delivery of alkaline metals, and the associated drawdown of atmospheric CO_2_ via silicate weathering (Berner et al., 1983) may have been underestimated from landscapes with significant glacial cover (Blackburn et al., 2019). Since the export of ASi from glacial systems is large, this represents a significant unknown when quantifying subglacial chemical weathering budgets and determining how variable glaciation could regulate the climate over glacial‐interglacial cycles (Blackburn et al., 2019; M. Torres et al., 2017).

### 4.2 Controls on Riverine Fe Concentrations and Composition

The low truly dissolved/soluble Fe (sFe; <0.02 μm) concentrations compared to colloidal‐nanoparticulate Fe (CNFe; 0.02–0.45 μm) and the lack of any significant trend with glacial cover (Figure 3a) are expected given the instability of truly dissolved Fe species in circumneutral, oxygenated waters and the low solubility of crystalline Fe(III) minerals (Raiswell & Canfield, 2012). The only river systems with elevated sFe concentrations in the sampling region were all organic‐rich rivers draining from humic soils or peat bogs, likely due to the effects of organic‐ligand stabilization of truly dissolved Fe species (Hopwood et al., 2014; Rose & Waite, 2003). For these organic‐rich river systems, ∼22% of the filterable iron (sFe + CNFe) was in the soluble phase, compared to 1.5% for organic‐poor rivers. This may be important because sFe is considered to be the best empirical measure of readily bioavailable Fe (Raiswell et al., 2018; Shaked & Lis, 2012; Tagliabue et al., 2017) and is more likely to (semi)conservatively mix with oceanic waters (Herzog et al., 2019, 2020; Oldham et al., 2017). Our data indicate that only a small proportion of Fe is in the sFe size fraction in rivers with lower dissolved organic carbon (DOC) concentrations, including those with a significant glacier meltwater component, and that organic Fe complexation is therefore not likely to be important for glacial Fe export. All Fe phases that can pass through a 0.22 or 0.45 μm pore‐size filter are nominally classed as “dissolved” in the majority of previous riverine studies and are mostly assumed to be labile. However, where colloidal‐size Fe‐bearing aluminosilicates and Fe‐oxyhydroxide nanoparticulate aggregates are present, this may be an incomplete assumption as labilities of Fe mineral and organic bound phases can differ greatly (Schroth et al., 2014; Shoenfelt et al., 2017). Furthermore, research has shown that the vast majority of CNFe species are removed during transport through estuarine environments due to flocculation and settling, meaning that these phases may not reach the open ocean (Boyle et al., 1977; Hopwood et al., 2015; Schroth et al., 2014).

CNFe phases account for a large proportion of the Fe budget in all Patagonian river systems, with concentrations 4–1,400 times higher than the sFe phase (Figure 3f). Glacier‐fed rivers have the highest CNFe concentrations in the region (mean = 5.43 ± 2.10 μM; Figure 3b), similar to CNFe and <0.45 μm filterable Fe concentrations reported from proglacial rivers in Alaska (7.75 ± 4.41 μM; Schroth et al., 2011), Peru (8.77 ± 7.84 μM; Fortner et al., 2011), and the Alps (6.95 μM; Mitchell et al., 2001). The linear increase in CNFe concentrations with increasing glacial cover in Patagonia indicates a subglacial origin for these phases, corresponding to the observation for CNSi. However, unlike for CNSi, CNFe phases were also found at detectable concentrations in all non‐glacial river systems, reflecting additional non‐glacier derived species. Our data indicate that the majority of CNSi is associated with the colloidal‐nanoparticulate feldspars that formed as a physical weathering product, whereas the CNFe fractions likely comprise a variety of crystalline and amorphous Fe phases, including complex mixtures of organic and mineral aggregates that may be secondary chemical weathering products (Raiswell et al., 2016, 2018). The EDX data suggest that the bulk composition of glacial colloidal‐nanoparticulate material can be as high as 12 wt.% Fe (supporting information Table S3), indicating that the majority of CNFe is not incorporated in feldspar minerals, which typically have very low Fe concentrations of <0.5 wt.% (Hofmeister & Rossman, 1984). Analyses of the mineralogy and phase speciation of Fe in glacial flour and glaciogenic dust (which are likely to have a similar composition to riverine CNFe) have shown that these phases are predominantly composed of Fe(II)‐bearing primary silicate minerals and clays, as well as poorly crystalline Fe oxyhydroxide nanoparticulate aggregates that are believed to be labile in downstream ecosystems (Hawkings et al., 2014, 2018; Raiswell et al., 2018; Schroth et al., 2009, 2011; Shoenfelt et al., 2017). However, the composition of glacial colloidal‐nanoparticulate material has never been directly analyzed before.

Despite testing the potential reactivity of the CNFe in Patagonian rivers and finding that roughly 15% of this fraction was ascorbate extractable, we highlight that the results of these experiments are not a full representation of the labile component for several reasons. First, we were only able to test one glacier‐fed river that had sufficient amounts of filtrate for analysis, meaning that our data will not reflect the full range of CNFe phases, which likely contain complex mixtures of different Fe components with variable labilities (Raiswell et al., 2018). Second, the colloidal‐nanoparticulate material was refiltered and extracted after ∼2.5 years storage at room temperature. Since ferrihydrite ages and transforms to more crystalline and less reactive iron species with a half‐life of ∼200 days (Raiswell & Canfield, 2012), our values likely represent a significant underestimate of the highly reactive Fe present at the time of sampling. Finally, refiltering samples through 0.22 μm filters will not have removed all colloidal‐nanoparticulate material from solution, despite flocculation during storage. Therefore, we cannot accurately approximate how much of the CNFe from Patagonian rivers is potentially reactive, representing a significant unknown in quantifying Fe exports from this region. Several lines of evidence, including EDX data showing ∼12 wt.% Fe and significant FeA concentrations despite aging and filtering issues, suggest that a large proportion of the CNFe may have originally been composed of highly reactive Fe (e.g., amorphous ferrihydrite). However, more research is needed to quantify the valence‐state, mineralogy and lability of CNFe phases (Raiswell et al., 2018), as well as constraining substantial modification of riverine exports in estuaries, and the fate of these phases in downstream ecosystems (Schroth et al., 2014).

The ascorbate extractable Fe concentrations (FeA; >0.45 μm) of the SPM load are highly variable in Patagonian rivers and account for a large proportion of reactive Fe exports. These FeA phases are thought to be mostly composed of freshly precipitated ferrihydrite, or Fe(II) adsorbed to silicate minerals, and are believed to represent the most bioavailable fraction of Fe associated with the SPM (Hawkings et al., 2014; Raiswell et al., 2016, 2018). The highest FeA (wt.%) values are exported from rivers draining from catchments with mafic bedrock, indicating that catchment lithology may play a role in determining FeA concentrations(Hartmann & Moosdorf, 2012). The relatively invariant FeA (wt.%) values from glacier‐fed rivers in this region suggest similar rates of production (via sulfide oxidation, silicate weathering, and/or iron reduction) beneath different glacial systems. The FeA (wt.%) concentrations from glacial rivers in Patagonia are roughly double previously reported values from Leverett Glacier in Greenland (0.15 ± 0.02 wt.%; Hawkings et al., 2014), perhaps due to differences in bedrock type, weathering rates or sediment grain‐size distribution and composition. In Patagonian rivers, concentration estimates of FeA (μM) can be derived from the SPM load, but catchments dominated by mafic bedrock deviate from the linear trend (Figure 3d). The high concentrations of FeA and the fact that this phase can dominate the Fe budget in high SPM load rivers demonstrates the need to include labile particulate fractions in terrestrial nutrient budgets. These sediment‐bound glaciogenic Fe phases have also been highlighted as more important than CNFe phases due to increased transportation through estuaries and fjords to the marine environment (Markussen et al., 2016; Schroth et al., 2014).

Recent research has also shown that some diatoms species can effectively “mine” and enhance dissolution of other particulate Fe phases that might not be quantified with the FeA extraction (Kessler et al., 2020; Rubin et al., 2011; Shoenfelt et al., 2017). For example, additions of Fe(II)‐rich glaciogenic dust from Patagonia have been shown to significantly enhance diatom growth in sFe‐limited culture experiments, compared to additions of Fe(III)‐rich non‐glaciogenic dust (Shoenfelt et al., 2017). These findings imply that Fe valence and operationally defined solubility may both be important for ascertaining the bioavailability of Fe species (Shoenfelt et al., 2017) and suggest that solely using FeA concentrations as a proxy for the “labile” fraction of particulate Fe could lead to significant underestimates of potentially bioavailable Fe exported from glaciated regions. As concluded for CNFe, more research is needed to quantify the lability and fate of particulate Fe species in order to fully resolve how glacial processes affect the Fe cycle and could modulate the climate system (Hawkings et al., 2018; Raiswell et al., 2018).

### 4.3 Influence of Glacial Cover on Si and Fe Yields in Chilean Patagonia

There have been few previous estimates of nutrient fluxes from Patagonian catchments to adjacent fjords, and so the data presented here represent the first insight into how glacial cover can affect weathering dynamics and the export of nutrients from this region. The high specific discharge rates from catchments with high glacial cover highlight the importance of glaciers as sources of fresh water in this region. However, there is also considerable variability between catchments, reflecting complex additional controls on specific discharge rates such as the surrounding topography, temperature, and rainfall patterns (Figure 5a). As such, the trends we observe in nutrient yields (i.e., fluxes normalized by upstream catchment area) are not primarily caused by the relationship between specific discharge and glacial cover. These yield calculations represent broad estimates, as they are not based on seasonally resolved data. However, we speculate that the large proglacial lakes in front of all the glacier‐fed river systems that were sampled will buffer and homogenize temporal and seasonal geochemical variability in exports. Therefore, our yield calculations from glacier‐fed rivers are likely to be broadly representative of average annual exports and not disproportionately biased toward summer melt‐season values. For non‐glacial river systems, we infer that temporal or seasonal variability will be less significant than differences between catchments, as shown by Vandekerkhove et al. (2016) from rivers in Patagonia.

The lack of relationship between glacial cover and sFe yields (Figure 5e) indicates that glacial weathering processes neither enhance nor depress sFe export from this region. Mirroring trends for concentrations, the yields of sFe are low and relatively invariant across the region, demonstrating that sFe species only accounts for a very small proportion of the total Fe budget, despite potentially representing the most bioavailable fraction (Raiswell et al., 2018; Shaked & Lis, 2012; Tagliabue et al., 2017). For DSi, the only catchments with significantly elevated DSi yield drained from active volcanoes. For all non‐volcanic catchments, the lack of a relationship between DSi yields and glacial cover indicates that glacial cover does not significantly affect DSi exports. This trend is likely due to decreased Si concentrations from catchments with more glacial cover having relatively high discharge rates due to significant inputs from melting glaciers. Indeed, these yield calculations may represent significant underestimates for highly glaciated catchments, as the entire catchment area was used rather than just the hydrologically active subglacial zone. As such, our data suggest that variable glacial cover does not significantly depress silicate weathering rates in Patagonia, contrasting long‐held assumptions about subglacial weathering budgets (Anderson et al., 1997; M. Torres et al., 2017; Tranter et al., 2002).

The significant positive linear relationships between glacial cover and the yields of colloidal‐nanoparticulate and reactive SPM‐bound Si and Fe indicate the importance of glaciers in enhancing total Si and Fe exports from Chilean Patagonia (Figures 5c–5f). For CNFe and CNSi, these trends are likely caused by increasing colloidal‐nanoparticulate concentrations with increasing glacial cover. For FeA and ASi, exports are controlled by the higher SPM yields, which increase as a function of glacial cover (Figure 5b). Yields of SPM and associated nutrient phases are roughly an order of magnitude higher in highly glaciated catchments than non‐glaciated catchments, highlighting how glaciers dramatically enhance physical weathering and the flux of sediment to downstream ecosystems (Koppes et al., 2015). Such large fluxes of SPM have complex implications for downstream ecosystems, providing substantial quantities of labile particulate nutrients such as Si, Fe, and other trace metals and also causing severe light limitation in near‐coastal environments and suppressing primary productivity (González et al., 2013; Hopwood et al., 2018; Murray et al., 2015).

### 4.4 Comparison to Si and Fe Exports From Other Glaciated Regions

We compare Si and Fe data from Patagonia to a global data set of size‐fractionated Si and Fe concentrations and yield estimates from other glacier‐fed rivers to contextualize the data presented here and to highlight potential gaps in research. Across the nine glaciated regions where data are available (Table 1), all except Svalbard have a relatively narrow range of mean DSi concentrations (24–45 μM), suggesting that rates of subglacial silicate weathering or supraglacial dilution are similar between regions. Svalbard exhibits significantly lower and invariant DSi concentrations compared to other regions, likely due to the combination of bedrock type and cold‐based thermal regimes reducing water‐rock interactions and therefore subglacial weathering (Hodgkins et al., 1997, 2002; Hodson et al., 2015). Mean DSi yields vary significantly between glaciated regions and are highest from Iceland, although these data are based on Si concentrations analyzed by ICP‐OES with DSi concentrations from 198–233 μM (Gislason et al., 1996; Hodson et al., 2000), which may include CNSi phases. Excluding Iceland and Svalbard, yields of DSi are highest from the Himalaya, Alaska, and Patagonia and lowest from Greenland, primarily reflecting variability in specific discharge rates.

**Table 1:** Compiled Global Si Concentration and Yield Data From Proglacial Rivers

	DSi	CNSi	ASi	ASi	Yield	Yield	Yield
	∗	0.02–0.45 μm	>0.45 μm	>0.45 μm	DSi	CNSi	ASi
Region	(μM)	(μM)	(wt.%)	(μM)	(Mg km^−2^ yr^−1^)	(Mg km^−2^ yr^−1^)	(Mg km^−2^ yr^−1^)
Patagonia^a^	32.0 ± 8.6	46.1 ± 28.5	0.57 ± 0.08	12.0 ± 4.3	4.57 ± 2.30	7.30 ± 6.00	1.67 ± 0.99
Greenland	25.4 ± 11.8^b^, ^c^, ^d^	—	0.62 ± 0.35^c^, ^d^	202 ± 191^c^, ^d^	1.21 ± 1.29^b^, ^c^, ^d^	—	22.0 ± 12.4^c^, ^d^
Alaska	23.5 ± 8.5^d^, ^e^	61.2 ± 67.6^f^	0.39 ± 0.21^g^	—	4.84 ± —^e^	—	—
Himalaya	44.5 ± 27.6^h^, ^i^	—	—	—	5.52 ± 4.41^h^, ^i^	—	—
Peru	—	—	—	—	—	—	—
Alps	27 ± 11^j^	—	—	—	2.00 ± 0.51^k^	—	—
Iceland	42.7 ± 32.8^g^	—	1.04 ± 0.82^g^	—	12.7 ± 2.3^l^, ^m^	—	—
Svalbard	3.8 ± 0.6^g^, ^n^, ^o^	—	0.10 ± 0.02^g^	—	0.23 ± 0.11^n^, ^o^	—	—
Antarctica	29.2 ± 8.9^p^	—	—	—	—	—	—

*Note*. Values show the mean of all available data ±1*σ*. — = no available data. * = Truly dissolved DSi measured colourimetrically with the molybdate method. Some data are temporally resolved over a melt‐season and others represent spot samples.

a This study.

b Yde et al. (2014).

c Hawkings et al. (2017).

d Hatton, Hendry, Hawkings, Wadham, Kohler, et al. (2019).

e Anderson et al. (2003).

f Schroth et al. (2011).

g Hatton, Hendry, Hawkings, Wadham, Opfergelt, et al. (2019).

h Hodson et al. (2002).

i Singh et al. (2012).

j Lamb et al. (1995).

k Hosein et al. (2004).

l Gislason et al. (1996).

m Hodson et al. (2000).

n Hodgkins et al. (1997).

o Hodson et al. (2002).

p Green et al. (2005).

Size‐fractionated sFe (<0.02 μm) and CNFe (0.02–0.45 μm) concentrations have only been reported for glacial systems in Patagonia, Greenland, and Alaska, and all show that sFe phases account for <1% of filterable Fe phases (Table 2). This result further highlights the need for size‐fractionated sampling and to understand the speciation and lability of CNFe phases in downstream ecosystems. Concentrations and yields of filterable Fe (<0.45 μm; sFe + CNFe) vary by more than an order of magnitude between regions, likely reflecting complex variability in water residence times, subglacial geochemistry, and weathering rates (Li et al., 2019). Filterable Fe concentrations are highest from glacial systems in Peru, Alaska, the Alps, and Patagonia, all of which have concentrations more than an order of magnitude higher than mean global riverine values (0.48 μM; Gaillardet et al., 2014). Such high Fe concentrations highlight that glacial systems are important components of the Fe cycle and that further research is needed to understand how Fe cycling will change in a warming world (Li et al., 2019). However, as discussed previously, details of the mineralogy, phase‐speciation and estuarine transformations are critical to determining the lability and impacts of CNFe in downstream ecosystems. We also speculate that some of the variability in regional filterable Fe concentrations could be caused by the variety of different filtering techniques used between studies, which may allow variable amounts of colloidal‐nanoparticulate material to pass into the filtrate and obscure the real environmental drivers of glacial Fe concentrations (Hall et al., 1996; Horowitz et al., 1992; Morrison & Benoit, 2001). For example, concentrations of 0.45 μm Fe within a single river water sample were found to vary by almost an order of magnitude depending on the type of filter membrane used (Horowitz et al., 1992). This issue has never been investigated for glacial rivers where colloidal‐nanoparticulate phases dominate and filtering artifacts may be even more pronounced.

**Table 2:** Compiled Global Fe Concentration and Yield Data From Proglacial Rivers

	sFe	CNFe	sFe+CNFe	FeA	FeA	Yield	Yield
	<0.02 μm	0.02–0.45 μm	<0.45 μm	>0.45 μm	>0.45 μm	sFe+CNFe	FeA
Region	(μM)	(μM)	(μM)	(wt.%)	(μM)	(Mg km^−2^ yr^−1^)	(Mg km^−2^ yr^−1^)
Patagonia^a^	0.02 ± 0.04	5.43 ± 2.10	5.45 ± 2.09	0.31 ± 0.09	3.36 ± 1.04	1.52 ± 0.72	0.97 ± 0.54
Greenland	0.007 ± 0.009^b^	0.70 ± 0.65^b^	0.71 ± 0.66^b^	0.15 ± 0.02^b^	29 ± —^b^	0.14 ± —^b^	5.78 ± —^b^
Alaska	0.005 ± 0.004^c^	7.75 ± 4.41^c^	7.75 ± 4.41^c^	—	—	0.82 ± 0.91^d^	—
Himalaya	—	—	1.18 ± 0.70^d^	—	—	0.20 ± 0.12^d^	—
Peru	—	—	8.77 ± 7.84^e^	—	—	3.14 ± 2.80^d^	—
Alps	—	—	6.96 ± —^f^	—	—	1.70 ± —^d^	—
Iceland	—	—	0.46 ± 0.33^g^, ^*^	—	—	0.12 ± 0.08^d^, ^*^	—
Svalbard	—	—	0.51 ± 0.21^j^, ^h^	—	—	0.06 ± 0.02^d^	—
Antarctica	—	—	0.65 ± 0.39^j^, ^k^	—	—	0.04 ± 0.04^k^	—

*Note*. Values show the mean of all available data ±1*σ*; — = no available data. Some data are temporally resolved over a melt‐season, and others represent spot samples.

a This study.

b Hawkings et al. (2014).

c Schroth et al. (2011).

d Li et al. (2019).

e Fortner et al. (2011).

f Mitchell et al. (2001).

g Galeczka et al. (2014).

* Concentrations and yield estimates from samples filtered through 0.2 μm filters.

h Zhang et al. (2015).

i Hodson et al. (2016).

j Green et al. (2005).

k Hodson et al. (2017).

At present, ASi and FeA (μM) concentration and yield estimates only exist for the Greenland Ice Sheet (Hatton, Hendry, Hawkings, Wadham, Kohler, et al., 2019; Hawkings et al., 2014, 2017) and now the Patagonian ice fields. Both ASi and FeA (wt.%) values are relatively similar between these regions, potentially indicating that formation mechanisms and weathering rates are broadly uniform. However, when scaled to μM concentrations and yield estimates, values are 8–17 times higher from Greenland than Patagonia (Tables 1 and 2). These large differences in ASi and FeA exports are primarily caused by variability in proglacial riverine SPM loads, which are ∼20 times higher in proglacial rivers in Greenland than in Patagonia (Hawkings et al., 2017). An important factor explaining the differences in SPM loads (and the export of labile particulate phases) is likely to be the presence of large proglacial lakes in front of the vast majority of land‐terminating glaciers in Patagonia (Wilson et al., 2018). These large proglacial lakes act as effective traps for sediment and nutrients due to reduced water velocity enhancing particle aggregation and settling (Bogen et al., 2014; Liermann et al., 2012). Proglacial lakes are a key feature of the rapidly deglaciating landscape of Patagonia, with 1,226 new glacial lakes forming from 1986–2016, representing a 325 km^2^ increase in size (Wilson et al., 2018). These landscape changes have likely had significant impacts on the export of SPM and reactive particulate nutrients from this region. Indeed, the formation and expansion of proglacial lakes are not unique to Patagonia, with research along a section of western Greenland showing a 44% increase in the number of proglacial lakes and a 20% increase in lake area between 1987 and 2010 (Carrivick & Quincey, 2014). As such, we hypothesize that new and expanding proglacial lakes that may form as a result of progressive glacial retreat will dramatically reduce exports of SPM and associated nutrient phases from Greenland, with complex implications for marine ecosystems in the Arctic.

### 4.5 How Will Continued Glacial Retreat Affect Si and Fe Exports From Chilean Patagonia?

**Figure 6:**
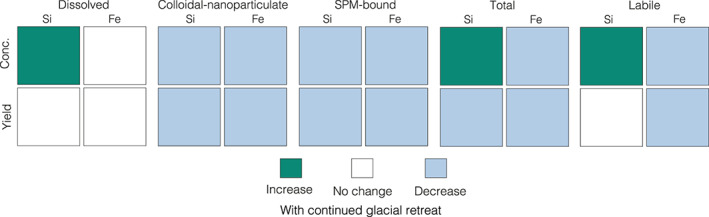
Conceptual schematic to show how regional‐scale riverine Si and Fe concentrations and yields in Chilean Patagonia are likely to change with declining glacial cover as inferred from the space‐for‐time substitution. Green shows increasing concentrations/yields, white shows no significant change and blue shows decreasing concentrations/yields with continued glacial retreat.

**Figure 7:**
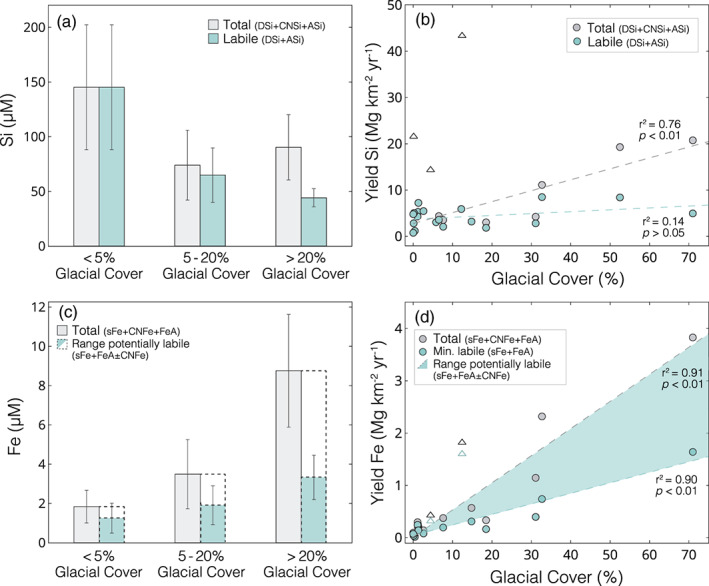
Total versus labile Si an Fe concentrations and yields. (a) Bar plots showing mean total‐Si (DSi + CNSi + ASi) and mean labile Si (DSi + ASi) for catchments categorized by glacial cover (%). Error bars show ±1*σ*. (b) Yield total (gray) and labile (green) Si versus glacial cover (%). (c) Bar plot showing mean total‐Fe (sFe + CNFe + FeA) and mean labile Fe for catchments categorized by glacial cover (%). The green‐shaded bar shows the lowest estimate of potentially labile‐Fe, (sFe, 0% CNFe, and FeA), and the dashed white bar shows the maximum, assuming that all CNFe is labile. Error bars show ±1*σ*. (d) Yield total (gray) and labile (green) Fe versus glacial cover. The green shaded area shows the potential difference in yields if some or all of the CNFe is labile. Bar plots exclude one volcano‐fed river (PTR01‐R23) due to disproportionately large SPM loads. Rivers draining from active volcanoes are shown with a triangle, and all other river systems are shown with a circle. Unfilled symbols are excluded from linear regression calculations.

Patagonia is currently experiencing rapid deglaciation, with widespread glacial retreat, the disappearance of small mountain glaciers, and the formation of new and expanding proglacial lakes (Dussaillant et al., 2019; Paul & Mölg, 2014; Wilson et al., 2018). Such dramatic landscape changes are likely to have profound impacts on terrestrial nutrient exports from this region. Applying space‐for‐time substitution, we use the current relationships with glacial cover to predict how Si and Fe exports will change with future glacial retreat in Chilean Patagonia (Figure 6). These inferences are based on long‐term (10^2^ to 10^3^ yr), regional‐scale trends observed with progressive deglaciation of a large area. We acknowledge that there are likely short‐term (years to decadal) perturbations in Fe and Si delivery that this analysis does not fully capture, for example, during the early phases of climate warming and glacier melt, when a temporary increase in glacial meltwater discharge (and solute yield) may occur despite a reduction in % glacier area (Foresta et al., 2018). These might be observed as fluctuations around the general trends observed in Figures 6 and 7 as glacier cover declines. A coupled model accounting for local‐scale hydrological change and temporal variability would allow for more nuanced projections in the future. However, as a thought experiment, these findings provide useful insights into whether regional‐scale exports of Si and Fe are likely to increase, decrease or experience no significant change with future glacial retreat, allowing for predictions of how deglaciation will impact the ecosystems surrounding Chilean Patagonia.

Despite decreasing riverine concentrations of CNSi and ASi with lower glacial cover in Patagonia, DSi concentrations increase with decreasing glacial cover and dominate the Si budget in non‐glacial rivers (Figure 2f). Assuming that these relationships hold into the future, our findings suggest that total Si concentrations (DSi + CNSi + ASi) are likely to increase with future glacial retreat. This trend is more pronounced when CNSi phases are excluded to give an indication of the labile Si budget (i.e., DSi + ASi), with labile Si concentrations roughly three times higher in non‐glaciated compared to highly glaciated catchments. As such, we hypothesize that labile riverine Si concentrations are likely to increase with continued glacial retreat across Patagonia. Conversely, the positive linear trend between total Si yields and glacial cover (for non‐volcano‐fed catchments) implies that total Si yields are likely to decrease with continued glacial retreat. This relationship is predominately due to decreasing concentrations of CNSi and ASi (which dominate Si budgets for glacier‐fed rivers) combined with overall trends of declining specific discharge in catchments with less glacial cover. However, when the CNSi is excluded from yield calculations due to its unconstrained reactivity in seawater, there is no significant trend between labile Si yields and glacial cover, indicating that regional‐scale exports of reactive Si are unlikely to change with continued glacial retreat (Figure 6 and 7b). The differences between total and labile Si yield estimates demonstrate the significance of accounting for CNSi components in glacier‐fed rivers in Patagonia. If the CNSi phases are even partially labile in seawater, the yields of reactive Si could significantly decrease with continued glacial retreat, with potential implications for downstream Si inventories and, by association, siliceous primary productivity.

Fe export trends are different from those of Si with future glacial retreat. The decreasing concentrations of CNFe and FeA with decreasing glacial cover and the negligible sFe component in most rivers result in mean total Fe (sFe + CNFe + FeA) concentrations that decrease almost fivefold from highly glaciated catchments to non‐glaciated catchments (Figure 7c and supporting information Table S2). The yields of total Fe are also roughly an order of magnitude greater from highly glaciated catchments than non‐glaciated catchments, excluding volcano‐fed riverine systems (Figure 7d). These relationships suggest that total riverine Fe concentrations and yields are likely to significantly decrease with continued glacial retreat. However, as the reactivity/fate of the CNFe phase is uncertain and likely to be complex, we leave the contribution of these phases to labile Fe budgets unconstrained. Further research is needed to quantify the composition and lability of CNFe phases in downstream ecosystems to understand the potential magnitude of change in labile riverine Fe exports with continued glacial retreat in Patagonia. As the extent of Fe limitation has not been investigated in the waters surrounding Chilean Patagonia, the full ecological impacts of reducing riverine Fe exports with future deglaciation are uncertain. However, as Fe‐depleted subantarctic surface water transported via the Antarctic Circumpolar Current has been shown to become trapped in Chilean coastal waters and enter the Patagonian fjords (Chaigneau & Pizarro, 2005; R. Torres et al., 2011), we speculate that terrestrial Fe exports are likely to be important for sustaining regional productivity. Overall, these data show that the concentrations, phase composition, and yields of both Si and Fe will change with continued glacial retreat across Patagonia, with implications for coastal ecosystems.

## 5 Conclusions

Concentrations of dissolved, colloidal‐nanoparticulate, and reactive SPM bound Si and Fe were measured in 40 river systems in Chilean Patagonia. Using the spectrum of glacial cover across this region, we investigate how glacial processes affect nutrient concentrations and yields, as well as inferring how regional‐scale exports are likely to change with continued deglaciation. We find that glacial cover impacts riverine concentrations, phase speciation and yields of both Si and Fe. The low DSi concentrations in all glacier‐fed rivers in Patagonia and the inverse relationship observed with glacial cover suggest that riverine DSi concentrations are likely to increase with declining glacial cover in this region. However, the similarity in DSi yield estimates between river catchments indicates that silicate weathering rates may not be reduced beneath glaciers and that regional DSi exports are unlikely to significantly change with continued glacial retreat. Both sFe concentrations and yields also showed no trend with glacial cover, indicating that glacial processes neither enhance nor depress sFe export. In contrast, the concentrations and yields of both CNSi and CNFe were roughly an order of magnitude greater from highly glaciated catchments and directly scaled with glacial cover. These colloidal‐nanoparticulate phases were predominately composed of finely ground feldspars formed from physical weathering beneath glaciers and are likely a common but compositionally heterogeneous component of riverine exports from mountainous glaciated catchments. The existence of high concentrations of colloidal‐nanoparticulate Fe and Si phases opens a discussion about the need for size‐fractionated analyses, standardized analytical protocols, and to quantify how variable filtering methods can affect measured concentrations. Future research should focus on understanding the environmental controls on subglacial colloid‐nanoparticulate formation, determining whether these phases are ubiquitous in glacier‐fed rivers around the world and constraining potential bioavailability in downstream ecosystems.

This study also highlights the need to account for reactive SPM bound phases of Si and Fe, both in terms of quantifying nutrient exports and resolving chemical weathering budgets. At present, FeA and ASi concentration and yield data have only been published from proglacial rivers in Greenland and Patagonia, potentially representing a significant gap in resolving glacial nutrient exports from other regions. In Patagonia, we demonstrate that FeA and ASi concentrations vary primarily as a function of the SPM load and that exports of these particulate nutrient phases are likely to significantly decline with continued glacial retreat and the formation of proglacial lakes. The impact of changes in SPM and particulate nutrient exports are likely to be spatially complex, significantly lowering bioavailable Si and Fe concentrations but also reducing light limitation in coastal ecosystems. Overall, we show the potential implications of declining glacial cover on terrestrial nutrient exports, helping to understand how glacial processes interact with biogeochemical cycles and may affect our changing climate.

## Conflict of Interest

The authors declare no conflicts of interest.

## Supporting information



Supporting Information S1

## Data Availability

All data used in this study are freely available to download (https://doi.org/10.5281/zenodo.3979589).
