# Supplementary material for: The Influence of Glacial Cover on Riverine Silicon and Iron Exports in Chilean Patagonia
Source: Global Biogeochem Cycles. 2020 Dec 17;34(12):e2020GB006611. doi: 10.1029/2020GB006611 (PMC7818384; doi:10.1029/2020GB006611)
Supplement: Supplementary file 1 — Supporting Information S1 [file GBC-34-e2020GB006611-s001.pdf]

Supplementary Information for 'The Influence of Glacial Cover on Riverine Silicon and Iron Exports in Chilean Patagonia'

Helena V. Pryer[1,2], Jon R. Hawkings[3,4], Jemma L. Wadham[1], Laura F. Robinson[2], Katharine R. Hendry[2], Jade E. Hatton[2], Anne M. Kellerman[3], Sebastien Bertrand[5], Beatriz Gill Olivas[1], Matthew G. Marshall[1], Richard A. Brooker[2], Giovanni Daneri[6,7], Vreni Häussermann[8].

[1]Bristol Glaciology Centre, Department of Geographical Sciences, University of Bristol
[2]School of Earth Sciences, University of Bristol
[3]Department of Earth, Ocean and Atmospheric Sciences, Florida State University, USA
[4]German Research Centre for Geosciences GFZ, Potsdam, Germany
[5]Renard Centre of Marine Geology, Ghent University, Ghent, Belgium
[6]Centro de Investigación en Ecosistemas de la Patagonia, Coyhaique, Chile
[7]COPAS Sur-Austral, Universidad de Concepción, Concepción, Chile
[8]Huinay Scientific Field Station, Pontificia Universidad Católica de Valparaíso, Valparaíso, Chile

Contents:

- Supplementary Text 1
- Supplementary Tables 1 – 5
- Supplementary Figure 1

Corresponding author: Helena Pryer, helena.pryer@bristol.ac.uk

Supplementary Text

1. Methods for colloidal-nanoparticulate extractions

The lability of colloidal-nanoparticulate silicon (CNSi) phases was tested by refiltering <0.45 µm filtered samples onto 0.22 µm Millipore polyethersulfone (PES) membrane filters, mounted onto swinnex filter holders ~30 months after sample collection. Refiltered volumes ranged from 10 – 50 ml and were limited by the volume of remaining sample. Six glacier-fed river samples were tested, although three produced results below the detection limit. These filters were then extracted in 20 ml of 0.1 M Na_2CO_3 at 85° C using the DeMaster (1981) method, as described in Section 2.3 of the main manuscript. Aliquots of the sample were collected after 2-, 3- and 5-hours and the Si concentrations were measured using flow injection analysis (FIA). The absorbance values were calibrated to seven standards and blank corrected to give Si concentrations in ppb. The y-intercept of the three-time points was calculated, representing the concentration of reactive amorphous silicon (ASi) associated with CNSi. These values were corrected to the volume of extractant and filtered sample, to give µM concentrations. These values were then compared to the total Si concentrations in the CNSi fraction, to give a percentage of the CNSi phases that was reactive ASi. For the three samples above the detection limit, values had a narrow range from 3.1 – 3.9%. As such, we assigned a conservative value of less than 5%, but it should be noted that not all samples were tested. These low values are to be expected since other data such as SEM-EDX and FT-IR-ATR suggest that the CNSi phase is predominately composed of feldspar and not ASi (Manuscript – Section 3.4).

The lability of colloidal-nanoparticulate iron (CNFe) phases was tested by refiltering 50 ml of <0.45 µm filtered samples onto 0.22 µm Millipore PES filters, mounted onto swinnex filter holders ~33 months after sample collection. Only one sample could be tested due to insufficient sample volumes for all other sites. This sample was the river with the most glacial cover (PFC01-SA) and was measured in triplicate, although the sample alliquots were from different days. The filters were then extracted in 5 ml of ascorbic acid solution for 24 hours using protocols developed by Raiswell et al. (2010) and described in more detail in Section 2.4 of the manuscript. Ascorbate-extractable iron (FeA) concentrations were then analysed with a Thermo Scientific Gallery Discrete Analyser using the ferrozine colourimetric method as outlined by Viollier et al. (2000). Concentrations were calibrated to seven standards of known concentrations and corrected for the volume of ascorbate-solution and the volume of sample filtered to give ascorbate-extractable iron concentrations of the CNFe phase. These values were then compared to the total Fe in the CNFe phase, as measured by ICP-OES and described in Section 2.4 of the manuscript, to give a percentage of the CNFe phase that is FeA and thus potentially labile. Values from these tests indicated that 12.4 – 15.8% of the CNFe phase was FeA.

For both CNSi and CNFe, it should be noted that there were several caveats with the methods used to test the lability of the colloidal-nanoparticulate phases. First, samples were refiltered through 0.22 µm filters, meaning not all of the colloidal-nanoparticulate material may have been removed, as we define the colloidal-nanoparticulate phase to include particles as small as 0.02 µm. For CNSi, we believe this may not represent a significant error, as the aluminosilicate grains had flocculated significantly during sample storage and the Si concentrations of the <0.22 µm fraction were within error of DSi concentrations from the FIA. However, for CNFe, the relative proportions that were removed by the 0.22 µm filter are unknown, meaning that our percentage FeA values may be a significant under-estimates. Second, the methods described above have

never been used to test colloidal-nanoparticulate phases and are calibrated for use on ~30 mg of suspended sediment (DeMaster, 1981; Raiswell et al., 2010). The sample weights used during these tests were very low (<1 mg), and the material is extremely fine-grained, meaning that the extractions potentially also remove additional phases and not just ASi and FeA. As such, the values cited could potentially be over-estimates of the proportions of these components in the colloidal-nanoparticulate fraction. Additionally, only three glacial river samples were tested for CNSi lability, and only one glacial river sample was tested for FeA. As such, these values likely do not reflect the full range of river samples collected during this campaign, as the composition of the colloidal-nanoparticulate phase is likely to be compositionally variable. Finally, the 0.45 μm filtered samples had been stored for ~2.5 years before refiltration. For CNSi, we hypothesise that this storage is unlikely to affect concentration or lability, as dissolved Si (DSi) concentrations were stable 1 – 18 months after collection, showing no gradual dissolution or precipitation of CNSi phases. However, since ferrihydrite is known to age and transform to less reactive species over time (Raiswell et al., 2016, 2018), the storage of these samples is likely to have dramatically reduced the FeA component of the CNFe phase if ferrihydrite was present at the time of sample collection. As such, the values of FeA given for CNFe may significantly under-estimate the labile fraction. Overall, we highlight that the lability of colloidal-nanoparticulate phases is uncertain and that further tests are needed to understand how this fraction is likely to react in downstream ecosystems.

Supplementary Table 1: Compiled geospatial and geochemical data from all river sampling locations. * = Below limit of detection; – = No data available; Dominant lithology using geological maps and classification system from Hartmann and Moosdorf (2012): pa = acid plutonic, mt = metamorphic, ss = siliciclastic sedimentary, vb = basic volcanic, py = pyroclastic, us = unconsolidated sediments.

Sample ID	Lat. (○)	Long. (○)	River Name	Date Sampled	Glacial Cover (%)	Catch. A (km^2)	Lithology (*)	SPM (mg L^{-1})	DSi (μM)	CNSi (μM)	ASi (wt. %)	ASi (μM)	sFe (nM)	CNFe (μM)	FeA (wt. %)	FeA (μM)
PTR01-R2	-42.4858	-72.3859	Río Vodudahue	2017-01-08	7.8	911	pa	3.5	67	*	1.12	1.4	8.6	0.53	3.31	2.24
PTR01-R4	-42.3624	-72.4038	Río Huinay	2017-01-09	1.2	108	mt	0.5	41	*	1.96	0.3	45.0	0.19	5.28	0.76
PTR01-R5	-42.9135	-72.7029	Río Chaiten	2017-01-10	0.1	61	pa	28.4	207	*	0.37	3.7	–	–	0.82	4.18
PTR01-R6	-42.9706	-72.7224	Río Yelco	2017-01-10	2.2	11354	pa	6.2	126	*	1.33	3.0	15.2	0.58	0.55	0.62
PTR01-R7	-43.1760	-72.4397	Lago Yelco	2017-01-10	1.7	10024	pa	2.8	138	*	1.86	1.9	145.2	0.74	0.18	0.32
PTR01-R8	-43.9908	-72.5050	Río Palena	2017-01-10	2.6	12506	pa	4.6	87	*	1.72	2.8	5.7	0.50	0.60	0.67
PTR01-R9	-44.4620	-72.5767	Río Ventisqueros	2017-01-10	32.7	176	pa	22.4	44	15.2	0.66	5.3	91.3	4.60	0.41	2.06
PTR01-R10	-44.6692	-72.4456	Río Cisnes A	2017-01-11	1.1	3607	pa	4.7	78	*	0.97	1.6	22.6	0.48	2.70	2.27
PTR01-R13	-44.6422	-72.2798	Río Grande	2017-01-12	12.2	126	pa	5.1	41	*	1.08	1.9	75.9	0.55	–	–
PTR01-R14	-44.6841	-72.2473	Río Cisnes B	2017-01-12	0.4	3213	pa	1.9	103	*	2.62	1.8	22.2	0.52	2.09	0.70
PTR01-R15	-45.0119	-72.1377	Río Maniguales	2017-01-12	0.6	335	pa	19.3	131	*	1.15	7.9	19.7	0.70	0.63	2.19
PTR01-R16	-45.1657	-72.1128	Río Nireguao	2017-01-12	0.1	1964	ss	8.6	176	*	2.09	6.4	–	–	0.83	1.27
PTR01-R17	-45.4029	-72.6505	Río Aysen	2017-01-13	1.3	11328	pa	5.8	167	*	1.85	3.8	66.1	0.85	1.56	1.62
PTR01-R18	-45.4308	-72.5874	Río Blanco	2017-01-13	4.4	2987	pa	7.3	171	*	1.44	3.7	21.0	0.68	2.12	1.92
PTR01-R19	-45.3987	-72.4752	Río Manihuales	2017-01-13	0.2	4333	pa	5.9	152	*	1.44	3.0	26.1	0.55	–	–
PTR01-R20	-45.5871	-72.0832	Río Simpson	2017-01-14	0.0	2410	vb	7.2	260	*	1.89	4.8	2.7	0.64	1.56	2.00
PTR01-R21	-45.8083	-71.9238	Río Blanco	2017-01-14	0.0	1675	vb	6.8	189	*	1.48	3.6	–	–	–	–
PTR01-R22	-46.1048	-72.1470	Estero Del Bosque	2017-01-14	14.2	33	py+us	2.9	86	*	–	–	–	–	–	–
PTR01-R23	-46.1333	-72.4191	Río Ibanez	2017-01-15	12.4	899	pa	240.9	248	*	0.66	56.8	10.4	0.77	0.13	5.65
PTR01-R24	-46.2292	-72.8089	Río Murta	2017-01-15	12.6	345	pa	22.8	99	*	0.55	4.5	4.6	3.14	0.56	2.30
PTR01-R25	-46.4576	-72.7215	Río Engano	2017-01-15	12.7	299	pa	8.3	56	*	0.67	2.0	–	–	0.8	1.18
PTR01-R26	-46.7372	-72.8600	Río Leones	2017-01-16	31.1	821	pa	70.2	36	24.8	0.50	12.4	7.1	6.50	0.27	3.44
PTR01-R27	-47.0795	-72.7791	Río Baker – L. Bert.	2017-01-16	5.8	15934	py	4.5	93	*	2.09	3.3	–	–	0.13	0.10
PTR01-R28	-47.1309	-72.7320	Río Baker – Nef.	2017-01-16	6.5	16732	py	62.8	82	21.5	0.64	14.4	–	–	0.22	2.48
PTR01-R29	-47.1163	-72.6070	Río Chacabuco	2017-01-16	1.6	1495	py	89.6	88	*	0.51	16.1	–	–	0.43	6.84
PTR01-R30	-47.3326	-72.6575	Río del Salto	2017-01-17	12.9	1030	mt	12.0	51	*	1.70	7.3	4.5	0.89	0.36	0.77
PTR01-R31	-47.5941	-72.8482	Río de los Nadis	2017-01-17	17.0	575	mt	23.7	36	*	0.41	3.4	–	–	0.32	1.37
PTR01-R32	-47.7217	-73.1721	Río Del Paso	2017-01-17	14.8	129	mt	5.0	31	*	–	–	–	1.54	0.36	0.32
PTR01-R33	-47.7899	-73.3845	Río Baker – Tortel	2017-01-17	7.6	29107	mt+py+pa	60.2	61	51.3	0.56	12.0	11.9	3.17	0.32	3.46
PTR01-RB	-47.9570	-73.1925	Río Bravo	2017-02-17	14.8	1062	mt	18.1	28	*	1.00	6.4	290.6	1.39	0.43	1.39
PTR01-RP	-48.2160	-73.3304	Río Pascua	2017-02-23	18.5	15079	mt	35.7	38	28.1	0.41	5.3	*	2.04	0.31	1.97
PTR02-TR2.1	-47.6642	-72.9865	Estero Lago Vargas	2017-01-22	11.7	85	mt	–	48	*	–	–	–	–	–	–
PTR02-TR2.2	-46.8772	-72.7892	Lago Carrera	2017-01-22	5.1	14845	py	–	102	*	–	–	–	–	–	–
PTR02-TR2.4	-44.4669	-72.5424	Lago Colgante	2017-01-24	35.1	166	pa	–	27	31.3	–	–	–	–	–	–
PTR02-TR2.8	-43.2809	-72.4528	Estero el Ventisquero	2017-01-24	50.1	25	vb	–	37	60.0	–	–	–	–	–	–
PTR03-S03	-46.5004	-73.1495	Río Exploradores	2017-10-26	52.5	193	pa	81.6	37	69.9	0.57	16.6	–	–	0.25	3.61
PFC01-SA	-47.5439	-73.6860	Río Huemules A	Jan – Feb 2017	71.0	671	pa	87.5	15	98.9	0.50	15.5	4.9	6.89	0.28	5.16
PFC01-SC	-47.5777	-73.6743	Río Huemules B	Jan – Feb 2017	56.5	1044	pa	74.6	30	44.7	0.51	13.6	5.2	7.05	0.23	3.06
PFC01-SGT	-47.5420	-73.6849	Glacial tributary	Jan – Feb 2017	38.7	242	pa	35.8	30	23.7	0.68	8.7	13.0	2.10	0.44	2.80
PFC01-SH	-47.5936	-73.6770	Río Quincey	Jan – Feb 2017	3.6	63	pa	1.0	35	*	–	–	140.8	0.56	1.29	0.22

Supplementary Table 2: Compiled geospatial and yield estimate data from all river sampling locations where discharge data was available. Discharge data sourced from Dirección General de Aguas database (http://snia.dga.cl/BNAConsultas/reportes), unless cited otherwise. Discharge values are mean annual data complied from all available records. Yield estimates in units of Mg km^{-2} year $^{-1}$; – = No data available.

Sample ID	Lat. (○)	Long. (○)	River Name	Glacial Cover (%)	Catch. A (km^2)	Mean A. Discharge (m^{3}s^{-1})	Specific Discharge (m yr^{-1})	Yield SPM (Mg km^{-2} yr^{-1})	Yield DSi (Mg km^{-2} yr^{-1})	Yield CNSi (Mg km^{-2} yr^{-1})	Yield ASi (Mg km^{-2} yr^{-1})	Yield sFe (Mg km^{-2} yr^{-1})	Yield CNFe (Mg km^{-2} yr^{-1})	Yield FeA (Mg km^{-2} yr^{-1})
PTR01-R2	-42.4858	-72.3859	Río Vodudahue	7.8	911	–	–	–	–	–	–	–	–	–
PTR01-R4	-42.3624	-72.4038	Río Huinay	1.2	108	15.4§	4.52	2.05	5.24	–	0.04	0.011	0.05	0.19
PTR01-R5	-42.9135	-72.7029	Río Chaiten	0.1	61	7.0	3.64	103.56	21.18	–	0.38	–	–	0.85
PTR01-R6	-42.9706	-72.7224	Río Yelco	2.2	11354	–	–	–	–	–	–	–	–	–
PTR01-R7	-43.1760	-72.4397	Lago Yelco	1.7	10024	–	–	–	–	–	–	–	–	–
PTR01-R8	-43.9908	-72.5050	Río Palena	2.6	12506	856.1	2.16	9.92	5.29	–	0.17	0.001	0.06	0.08
PTR01-R9	-44.4620	-72.5767	Río Ventisqueros	32.7	176	34.3	6.15	137.69	7.57	2.63	0.91	0.031	1.58	0.71
PTR01-R10	-44.6692	-72.4456	Río Cisnes A	1.1	3607	220.0	1.92	9.02	4.20	–	0.09	0.002	0.05	0.24
PTR01-R13	-44.6422	-72.2798	Río Grande	12.2	126	19.7	4.92	24.95	5.63	–	0.27	0.021	0.15	
PTR01-R14	-44.6841	-72.2473	Río Cisnes B	0.4	3213	40.4	0.40	0.75	1.15	–	0.02	0.000	0.01	0.02
PTR01-R15	-45.0119	-72.1377	Río Maniguales	0.6	335	–	–	–	–	–	–	–	–	–
PTR01-R16	-45.1657	-72.1128	Río Nireguao	0.1	1964	34.1	0.55	4.68	2.71	–	0.10	–	–	0.04
PTR01-R17	-45.4029	-72.6505	Río Aysen	1.3	11328	539.9	1.50	8.71	7.07	–	0.16	0.006	0.07	0.14
PTR01-R18	-45.4308	-72.5874	Río Blanco	4.4	2987	276.3	2.92	21.22	14.04	–	0.31	0.003	0.11	0.31
PTR01-R19	-45.3987	-72.4752	Río Manihuales	0.2	4333	158.5	1.15	6.79	4.94	–	0.10	0.002	0.04	
PTR01-R20	-45.5871	-72.0832	Río Simpson	0.0	2410	49.3	0.65	–	4.71	–	0.09	0.000	0.02	0.07
PTR01-R21	-45.8083	-71.9238	Río Blanco	0.0	1675	7.4	0.14	0.95	0.74	–	0.01	–	–	–
PTR01-R22	-46.1048	-72.1470	Estero Del Bosque	14.2	33	–	–	–	–	–	–	–	–	–
PTR01-R23	-46.1333	-72.4191	Río Ibanez	12.4	899	144.4	5.07	1220.60	35.27	–	8.08	0.003	0.22	1.60
PTR01-R24	-46.2292	-72.8089	Río Murta	12.6	345	–	–	–	–	–	–	–	–	–
PTR01-R25	-46.4576	-72.7215	Río Engano	12.7	299	–	–	–	–	–	–	–	–	–
PTR01-R26	-46.7372	-72.8600	Río Leones	31.1	821	53.7	2.06	144.84	2.09	1.44	0.72	0.001	0.75	0.40
PTR01-R27	-47.0795	-72.7791	Río Baker – L. Bert	5.8	15934	568.2	1.13	5.07	2.95	–	0.11	–	–	0.01
PTR01-R28	-47.1309	-72.7320	Río Baker – Nef	6.5	16732	699.2	1.32	82.76	3.04	0.80	0.53	–	–	0.18
PTR01-R29	-47.1163	-72.6070	Río Chacabuco	1.6	1495	–	–	–	–	–	–	–	–	–
PTR01-R30	-47.3326	-72.6575	Río del Salto	12.9	1030	–	–	–	–	–	–	–	–	–
PTR01-R31	-47.5941	-72.8482	Río de los Nadis	17.0	575	–	–	–	–	–	–	–	–	–
PTR01-R32	-47.7217	-73.1721	Río Del Paso	14.8	129	–	–	–	–	–	–	–	–	–
PTR01-R33	-47.7899	-73.3845	Río Baker – Tortel	7.6	29107	931.6	1.01	60.78	1.73	1.46	0.34	0.001	0.18	0.20
PTR01-RB	-47.9570	-73.1925	Río Bravo	14.8	1062	112.0+	3.33	60.13	2.59	–	0.60	0.054	0.26	0.26
PTR01-RP	-48.2160	-73.3304	Río Pascua	18.5	15079	714.4	1.50	53.45	1.62	1.18	0.22	0.000	0.17	0.16
PTR02-TR2.1	-47.6642	-72.9865	Estero Lago Vargas	11.7	85	–	–	–	–	–	–	–	–	–
PTR02-TR2.2	-46.8772	-72.7892	Lago Carrera	5.1	14845	–	–	–	–	–	–	–	–	–
PTR02-TR2.4	-44.4669	-72.5424	Lago Colgante	35.1	166	34.3	6.54	–	4.89	5.75	–	–	–	–
PTR02-TR2.8	-43.2809	-72.4528	Estero el Ventisquero	50.1	25	–	–	–	–	–	–	–	–	–
PTR03-S03	-46.5004	-73.1495	Río Exploradores	52.5	193	34.0†	5.55	452.60	5.82	10.89	2.58	–	–	1.12
PFC01-SA	-47.5439	-73.6860	Río Huemules A	71.0	671	121.0§	5.69	497.76	2.47	15.80	2.47	0.002	2.19	1.64
PFC01-SC	-47.5777	-73.6743	Río Huemules B	56.5	1044	–	–	–	–	–	–	–	–	–
PFC01-SGT	-47.5420	-73.6849	Glacial tributary	38.7	242	–	–	–	–	–	–	–	–	–
PFC01-SH	-47.5936	-73.6770	Río Quincey	3.6	63	–	–	–	–	–	–	–	–	–

§ = Rhodamine Trace; + = Pantoja et al. (2011); † = Data digitised from Aniya et al. (2011)

Supplementary Table 3: Mean and standard deviation of riverine Si concentrations when grouped into three categories of glacial cover. The statistical significance of values between categories was tested using a single factor analysis of variance (ANOVA). Total Si = DSi + CNSi + ASi; Labile Si = DSi + ASi. Values exclude sample PTR01-R23, which drained from a volcano and had disproportionately high SPM concentrations.

	**DSi** (μM)		**CNSi** (μM)		**ASi** (μM)		**Total Si** (μM)		**Labile Si** (μM)	
Glacial Cover	Mean	σ	Mean	σ	Mean	σ	Mean	σ	Mean	σ
< 5 %	134.3	60.9	0.0	0.0	4.3	3.8	145.2	57.1	145.2	57.1
5 – 20 %	61.3	25.5	6.7	15.1	5.6	4.2	74.0	31.9	64.9	24.9
> 20 %	32.7	8.6	46.1	28.5	12.0	4.3	90.3	29.8	44.1	8.3
ANOVA	$F_2 = 19.1$ $p < 0.001$		$F_2 = 25.1$ $p < 0.001$		$F_2 = 8.1$ $p < 0.01$		$F_2 = 8.5$ $p < 0.001$		$F_2 = 17.5$ $p < 0.001$	

Supplementary Table 4: Mean and standard deviation of riverine Fe concentrations when grouped into three categories of glacial cover. The statistical significance of values between categories was tested using a single factor analysis of variance (ANOVA). Total Fe = sFe + CNFe + FeA; Labile Fe = sFe + FeA (lowest estimate). Values exclude PTR01-R23, which drained from a volcano and had disproportionately high SPM concentrations.

	**sFe** (μM)		**CNFe** (μM)		**FeA** (μM)		**Total Fe** (μM)		**Labile Fe** (μM)	
Glacial Cover	Mean	σ	Mean	σ	Mean	σ	Mean	σ	Mean	σ
< 5 %	0.04	0.05	0.58	0.16	1.87	1.78	1.84	0.83	1.25	0.76
5 – 20 %	0.06	0.11	1.66	1.06	1.60	1.00	3.49	1.76	1.91	0.99
> 20 %	0.02	0.04	5.43	2.10	3.36	1.04	8.76	2.87	3.33	1.13
ANOVA	$F_2 = 0.3$ $p > 0.7$		$F_2 = 34.0$ $p < 0.001$		$F_2 = 3.2$ $p < 0.05$		$F_2 = 28.3$ $p < 0.001$		$F_2 = 8.7$ $p < 0.001$	

Supplementary Table 5: Colloidal-nanoparticulate composition data from the SEM-EDX of sample PFC01-SA (Ruemules), filtered onto a cellulose nitrate filter membrane. Elemental concentrations are given as wt. % values for individual scans, as well as a bulk mean value. These data are semi-quantitative and the EDX resolution is larger than the particle size, so scans likely represent composite signals. EDX values represent emissions from the K-shell series (-K) or the L-shell series (-L). The mean composition suggests 92% alkali feldspar with an average composition of $(Na_{0.6}K_{0.3}Ca_{0.1})AlSi_3O_8$, with remaining elements suggesting a Mg-rich Fe-oxide, such as magnetite.

	Elemental Concentration (Wt.%)								
**Scan #**	**O-K**	**Na-K**	**Mg-K**	**Al-K**	**Si-K**	**K-K**	**Ca-K**	**Fe-K**	**Ba-L**
1	46.8	6.4	0.4	12.0	27.2	1.0	5.0	1.1	0.0
2	45.0	2.5	1.9	9.5	24.5	3.2	3.3	8.8	0.4
3	44.5	1.8	3.6	9.8	23.6	6.3	0.0	9.6	0.0
4	44.1	2.4	4.5	10.3	21.6	2.7	1.3	12.0	0.8
5	47.3	8.5	0.5	10.0	29.0	1.3	1.9	1.1	0.0
6	47.5	8.7	0.3	10.4	29.6	0.6	1.7	1.3	0.0
7	43.1	1.9	6.3	3.0	22.8	0.8	0.0	12.8	0.0
8	45.9	1.6	0.6	9.7	29.3	11.4	0.0	1.6	0.0
9	46.8	7.3	0.1	11.4	27.7	0.6	4.0	1.3	0.0
10	48.7	2.4	1.7	5.6	34.4	2.2	1.7	3.4	0.0
11	45.9	1.5	0.5	9.6	29.5	12.0	0.0	1.0	0.0
12	47.0	3.1	0.8	8.4	30.7	3.3	1.9	3.4	0.4
13	46.5	2.4	0.9	8.4	29.1	4.0	3.0	4.2	0.0
14	46.7	3.0	0.6	8.2	29.9	4.0	1.6	4.3	0.0
**Mean**	46.1	3.8	1.6	9.0	27.8	3.8	1.8	4.7	0.1

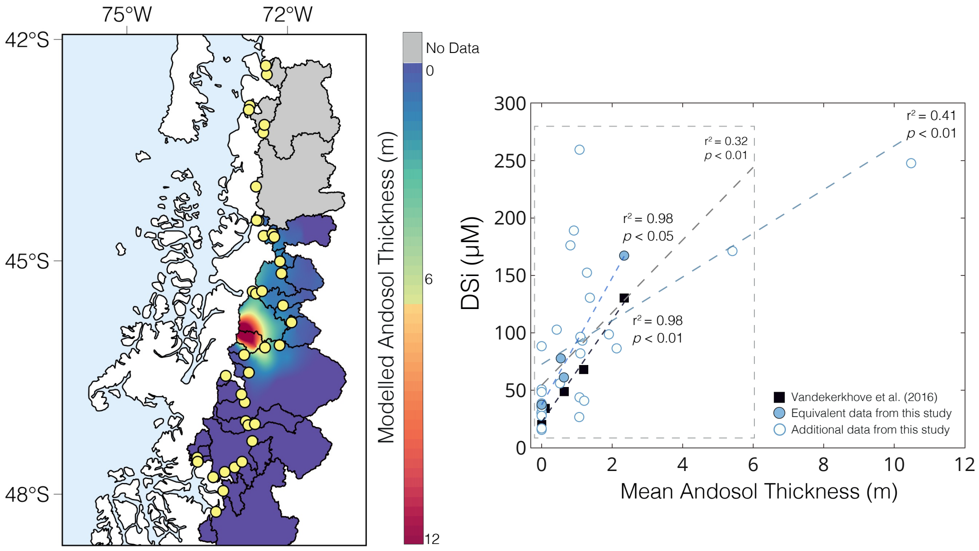

Supplementary Figure 1: Riverine DSi and Mean Andosol Thickness – **(a)** Map showing the modelled andosol thickness of river catchments measured as part of this study. This model was developed by Vandekerkhove et al. (2016) by interpolation of 68 soil profiles from across the region; **(b)** Scatter plot showing the relationship between mean andosol thickness in the upstream catchment area (m) and riverine DSi (μM). The data from Vandekerkhove et al. (2016) is shown with dark blue squares, showing a strong positive correlation (r^2 = 0.98, p < 0.01, n = 5). The same river catchments from our dataset are shown with filled light-blue circles and show the same strong positive correlation. (r^2 = 0.98, p < 0.05, n = 4). However, when we include all the additional samples measured during this study (non-filled light-blue circles), the relationship weakens (All data: r^2 = 0.41, p < 0.01, n = 36. Excluding the >10 m andosol thickness catchment: r^2 = 0.32, p < 0.01, n = 35).

References

Aniya, M., Skvarca, P., Sugiyama, S., Aoki, T., Matsumoto, T., Anma, R., ... Barcaza, G. (2011). Glaciological Research Project in Patagonia 2006-2009 Studies at Glaciar Perito Moreno, Hielo Patagónico Sur, in area of Hielo Patagónico Norte, and along the Pacific Coast. *Bulletin of Glaciological Research*, *29*, 1–17. doi: https://doi.org/10.5331/bgr.29.1

DeMaster, D. J. (1981). The supply and accumulation of silica in the marine environment. *Geochimica et Cosmochimica Acta*, *45*(10), 1715–1732. doi: https://doi.org/10.1016/0016-7037(81)90006-5

Hartmann, J., & Moosdorf, N. (2012). The new global lithological map database GLiM: A representation of rock properties at the Earth surface. *Geochemistry, Geophysics, Geosystems*, *13*(12), 119. doi: https://doi.org/10.1029/2012GC004370

Pantoja, S., Luis Iriarte, J., & Daneri, G. (2011). Oceanography of the Chilean Patagonia. *Continental Shelf Research*, *31*(3-4), 149–153. doi: https://doi.org/10.1016/j.csr.2010.10.013

Raiswell, R., Hawkings, J., Eisenousy, A., Death, R., Tranter, M., & Wadham, J. (2018). Iron in Glacial Systems: Speciation, Reactivity, Freezing Behavior, and Alteration During Transport. *Frontiers in Earth Science*, *6*, 222. doi: https://doi.org/10.3389/feart.2018.00222

Raiswell, R., Hawkings, J. R., Benning, L. G., Baker, A. R., Death, R., Albani, S., ... Tranter, M. (2016). Potentially bioavailable iron delivery by iceberg-hosted sediments and atmospheric dust to the polar oceans. *Biogeosciences*, *13*, 3887–3900. doi: https://doi.org/10.5194/bg-2016-20

Raiswell, R., Vu, H. P., Brinza, L., & Benning, L. G. (2010). The determination of labile Fe in ferrihydrite by ascorbic acid extraction: Methodology, dissolution kinetics and loss of solubility with age and de-watering. *Chemical Geology*, *278*(1), 70–79. doi: https://doi.org/10.1016/j.chemgeo.2010.09.002

Vandekerkhove, E., Bertrand, S., Reid, B., Bartels, A., & Charlier, B. (2016). Sources of dissolved silica to the fjords of northern Patagonia (44–48°S): the importance of volcanic ash soil distribution and weathering. *Earth Surface Processes and Landforms*, *41*(4), 499–512. doi: https://doi.org/10.1002/esp.3840

Viollier, E., Inglett, P. W., Hunter, K., Roychoudhury, A. N., & Van Cappellen, P. (2000). The ferrozine method revisited: Fe(II)/Fe(III) determination in natural waters. *Applied Geochemistry*, *15*(6), 785–790. doi: https://doi.org/10.1016/S0883-2927(99)00097-9
